# Kallikrein proteoforms and reproductive parameters in stallion are conditioned by climate

**DOI:** 10.1038/s41598-022-21350-w

**Published:** 2022-11-04

**Authors:** Renato Lima Senra, Camilo José Ramírez-López, Marcos Jorge Magalhães-Júnior, João Gabriel da Silva Neves, Edvaldo Barros, Bruna Waddington, Simone Eliza Facioni Guimarães, José Domingos Guimarães, Maria Cristina Baracat-Pereira

**Affiliations:** 1 Proteomics and Protein Biochemistry Laboratory, Universidade Federal de Viçosa, Viçosa, Brazil; 2 Animal Reproduction Laboratory, Universidade Federal de Viçosa, Viçosa, Brazil; 3 LABTEC-Animal Biotechnology Laboratory, Universidade Federal de Viçosa, Viçosa, Brazil; 4 Nucleus for Analysis of Biomolecules, Universidade Federal de Viçosa, Viçosa, Brazil

**Keywords:** Proteomics, Biochemistry

## Abstract

Horses are seasonal polyoestrous animals, and the photoperiod is the main factor modulating their reproductive activity. There is no consensus on the andrological and biochemical factors that influence breeding seasonality. To assess the involvement of climate in reproduction, Mangalarga Marchador stallions were monitored over 1 year regarding semen quality and seminal plasma proteome. Here, we show that kallikrein (KLKs) proteoforms in seminal plasma are involved in climate conditioning of reproduction. During the breeding season, greater abundance and different types of KLKs occurred simultaneously to lower sperm motility, greater semen volumes and higher concentrations of glucose and cholesterol. Considering that vasodilation due to activation of the kallikrein-kinin system and the consequent inhibition of the renin-angiotensin system may be associated with lower sperm motility, unravelling the involvement of KLK proteoforms in reproductive seasonality is a priority in horse breeding.

## Introduction

The reproductive seasonality in equines favours the return to a continuous cyclicity of females, making them more quickly pregnant during the breeding season^1^. Species of the genus *Equus* are seasonal polyoestrous animals, with the natural breeding season occurring from April to September in the Northern Hemisphere^1^ and from October to April in the Southern Hemisphere^2^. The differences in day length caused by bioclimatic changes conditionate the circannual rhythm of the reproductive activity^3^. Mangalarga Marchador, used in this work, is a typically Brazilian breed used in livestock and leisure, showing great economic importance in the national scenario^4^.

Although the reproductive seasonality in stallions is well known not only in the Mangalarga Marchador breed, the characterisation and determination of changes in reproductive parameters are not well defined. Assessments of the quality of fresh equine semen over the seasons in different breeds and countries do not show a consensus about the factors influencing seasonality. Whilst Blottner et al.^5^ reported seasonal variations in sperm morphology and motility, Wrench et al.^6^ found no differences for motility, number and concentration of sperm throughout the climatic seasons. Janett et al.^7^ found differences in the summer, such as higher values of volume, total number of spermatozoa and motility, with lower concentrations of normal spermatozoa. Morte et al.^8^ reported differences in spermatic concentration, vitality and morphology, but the progressive motility did not differ among seasons. Recently, Ebel et al.^9^ evaluated seasonal changes in the quality of sperm from fertile stallions, and only viability showed higher values during the breeding season; Johannisson et al.^10^ found minor variations in kinematics and morphology of sperm among seasons.

Seminal plasma, the fluid fraction of the semen, contains fundamental components for spermatic capacitation and fertility. The presence and amount of proteic and non-proteic biomolecules may indicate the quality of the semen^11^. In addition, a higher concentration of glucose than fructose in stallion semen, besides acting as an energetic substrate, also increases the resistance to membrane disorganisation and disruption^12^.

Seminal plasma proteins and factors surrounding them have been studied in different mammals, especially bulls^13–15^ and stallions^16–18^. Such proteins are involved in important stages of fertilisation, such as the establishment of sperm reserves in the oviduct, capacity modulation and interaction between gametes^10,16^. Identifying, characterising and quantifying seminal plasma proteins therefore allow inferences about the animal reproductive status.

Groups of proteins have been reported as associated with mammalian^19^ and stallion reproductive capacity, such as fibronectin type II modules, cysteine-rich secretory proteins (CRISPs) and spermadhesins^16^, albumins and kallikreins^9^. In horses, different semen proteins have been reported for the breeding and non-breeding seasons^9,10^. Few two-dimensional electrophoresis (2-DE) proteomics studies have added information about the mapping of seminal plasma proteoforms of mammals, including equines^18,20^. The current study aimed to disentangle the involvement of these proteoforms in stallion semen quality and to determine the influence of seasonality on equine reproduction through stallion semen and seminal plasma proteome characterisation throughout the climatic seasons.

## Results

### Reproductive seasonality

**Figure 1:**
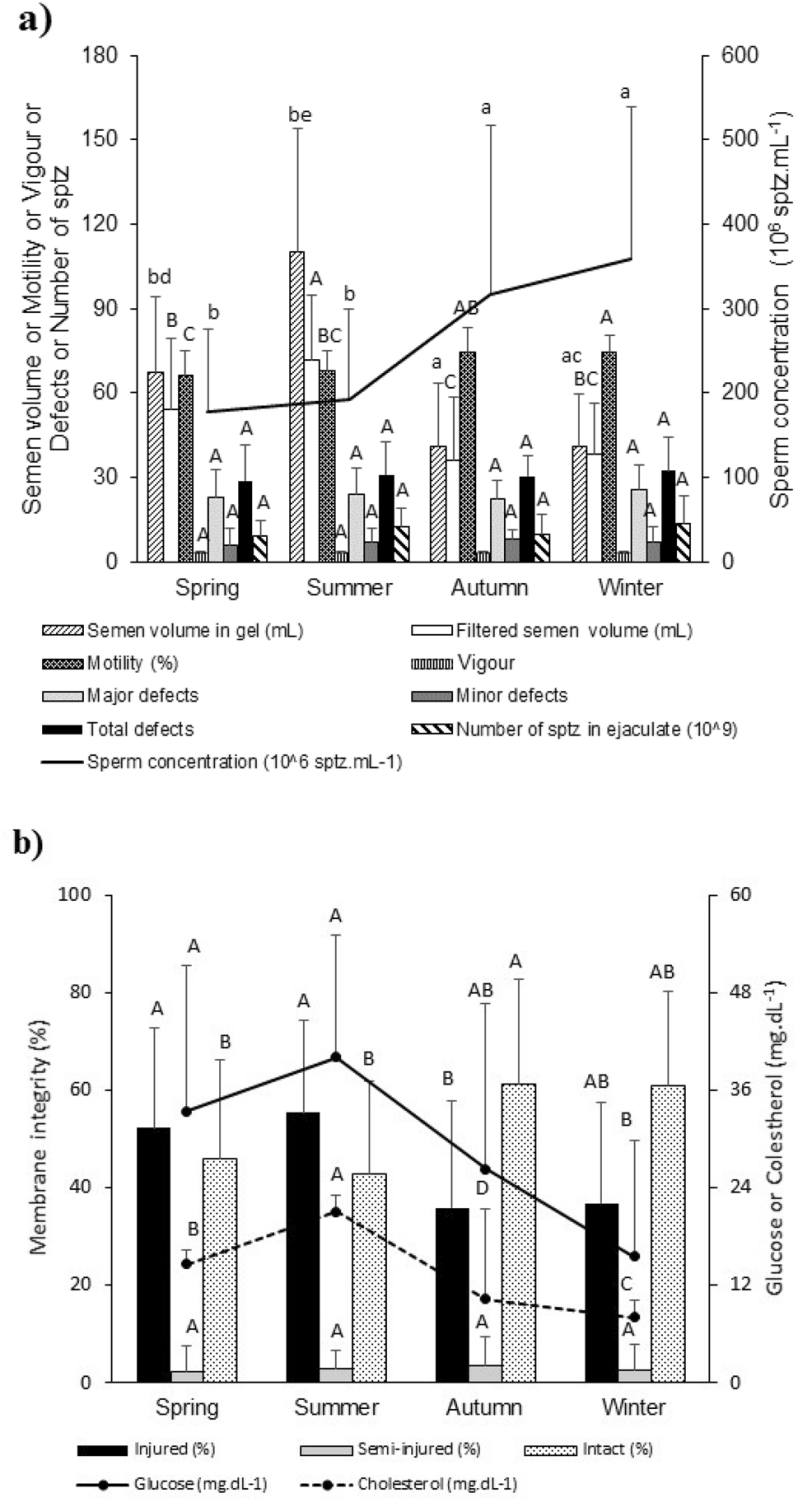
Morphological and physical–chemical parameters of stallion semen influencing reproductive seasonality. (**a**) Physical parameters of the semen of Mangalarga Marchador stallions throughout the four seasons of the year. Mean values followed by different lowercase letters on the same bar differ (*p* < 0.05) by the Kruskal Wallis test. Mean values followed by different capital letters on the same bar differ from each other (*p* < 0.05) by Tukey's test. Sptz means spermatozoa. (**b**) Concentrations of glucose and cholesterol in seminal plasma and evaluation of the plasma membrane integrity of Mangalarga Marchador stallion sperm throughout the four seasons. Bars accompanied by different capital letters differ from each other (*p* < 0.05) by Tukey's test.

Semen physical and morphological parameters were assessed for three stallions during the 4-year seasons (Fig. 1), in accordance with the determined climatological parameters (Supplementary Fig. 1), which showed different behaviours (*p* < 0.05) between the periods spring–summer (breeding season) and autumn–winter (sexual rest). Since the results from spring and summer were similar, as well as those from autumn and winter, two different reproductive behaviours were confirmed throughout the year, although no differences between summer and autumn were observed (Fig. 1a).

The Black Globe Temperature and Humidity Index (BGTHI) values and the luminous intensity presented by the luximeter differed (*p* < 0.05) between the periods of analysis in the stalls and paddocks. Besides, cortisol concentrations presented mean values below 2.8 μg dL^−1^ in the bloodstream of the stallions (Supplementary Fig. 1), indicating non-stressful conditions for the animals. The lowest (*p* < 0.05) cortisol concentration was observed in the summer, with the warmest and longest days of the year and the peak of the breeding season.

Regarding the evaluation of morphological and physical–chemical parameters, during the breeding season (spring–summer), there was about twice as much volume for semen in the gel and filtrate, as well as a lower sperm concentration per mL (*p* < 0.05), allowing a total sperm count similar in all seasons (Fig. 1a). Also, there was a significant positive Pearson correlation (r = 0.51, *p* < 0.05) between the means of light intensity (Supplementary Fig. 1) and the amount of gel in the ejaculate (Fig. 1a), which presented the highest values in the summer. Sperm motility showed a similar behaviour, differing (*p* < 0.05) between the winter and spring–summer seasons and between spring and autumn–winter (Fig. 1a). Regarding sperm vigour, defects (major, minor and total) and the number of spermatozoa in ejaculates, no difference (p > 0.05) was observed in mean values among the climatic seasons.

Blood plasma testosterone concentration (ranging from 0.41 to 0.55 ng mL^−1^) did not differ (*p* > 0.05) throughout the year. Also, soluble protein concentrations in the seminal plasma samples did not differ (p > 0.05) throughout the year, both for the Bradford (ranging from 12.25 to 15.92 mg mL^−1^) and the bicinchoninic acid method (BCA, ranging from 11.43 to 15.72 mg mL^−1^). Although the principles of protein determination are different for these methods, a high positive Pearson correlation value (r = 0.73) was found.

The mean values of glucose and cholesterol concentrations differed among the climatic seasons (*p* < 0.05) (Fig. 1b), according to the volume of semen produced during the year (Fig. 1a). In turn, the volume of semen produced did not correlate (Pearson's simple correlation) with any other studied characteristic (Fig. 1a), except for blood cortisol concentration (r = − 0.50, Supplementary Fig. 1); additionally, similarly to BGTHI and luminous intensity (Supplementary Fig. 1), glucose and cholesterol concentrations (Fig. 1b) were higher in spring–summer than in autumn–winter (Supplementary Fig. 2).

### Seminal plasma proteome in stallions

**Figure 2:**
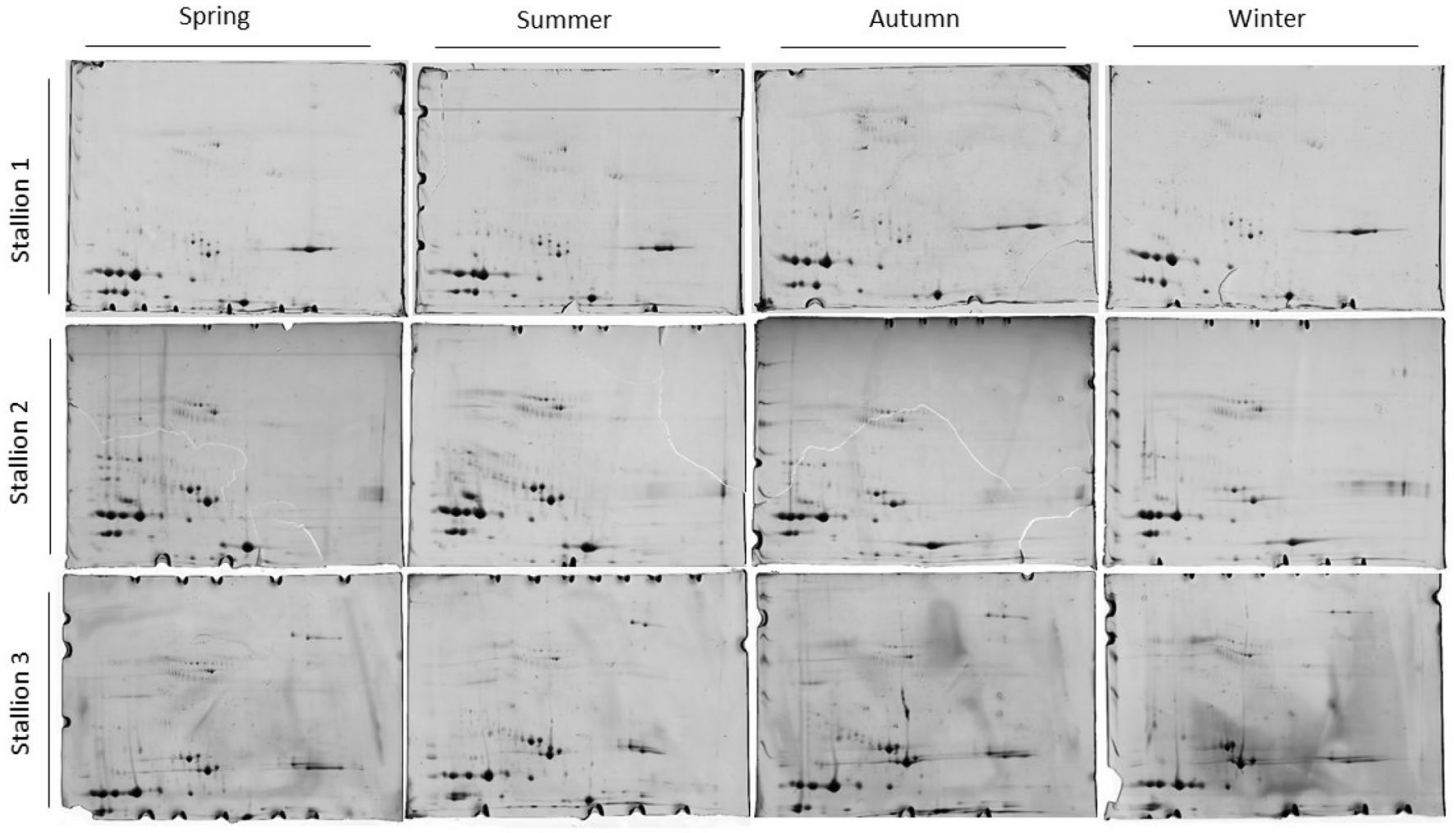
Two-dimensional electrophoresis gels of the seminal plasma of the stallions over the climatological seasons. A single gel for each animal and season was selected among the three technical replicates (Supplementary Figs. 2, 3, 4) to represent the protein profile of the treatment. The proteins were separated using pH 3–10 immobilised pH gradient (IPG) strips (24-cm, linear) and 12.5% T SDS-PAGE gels. Broad-Range Molecular Marker (Bio-Rad) was used (14.4–200 kDa).

The yearly seminal plasma protein profiles from three stallions, obtained by two-dimensional electrophoresis (2-DE), were similar but not identical (Fig. 2). Among the 36 two-dimensional gels analysed in this work (Supplementary Figs. 3, 4, 5), a 2-DE gel from each animal and season was selected to represent the protein profile of each treatment (Fig. 2).

**Figure 3:**
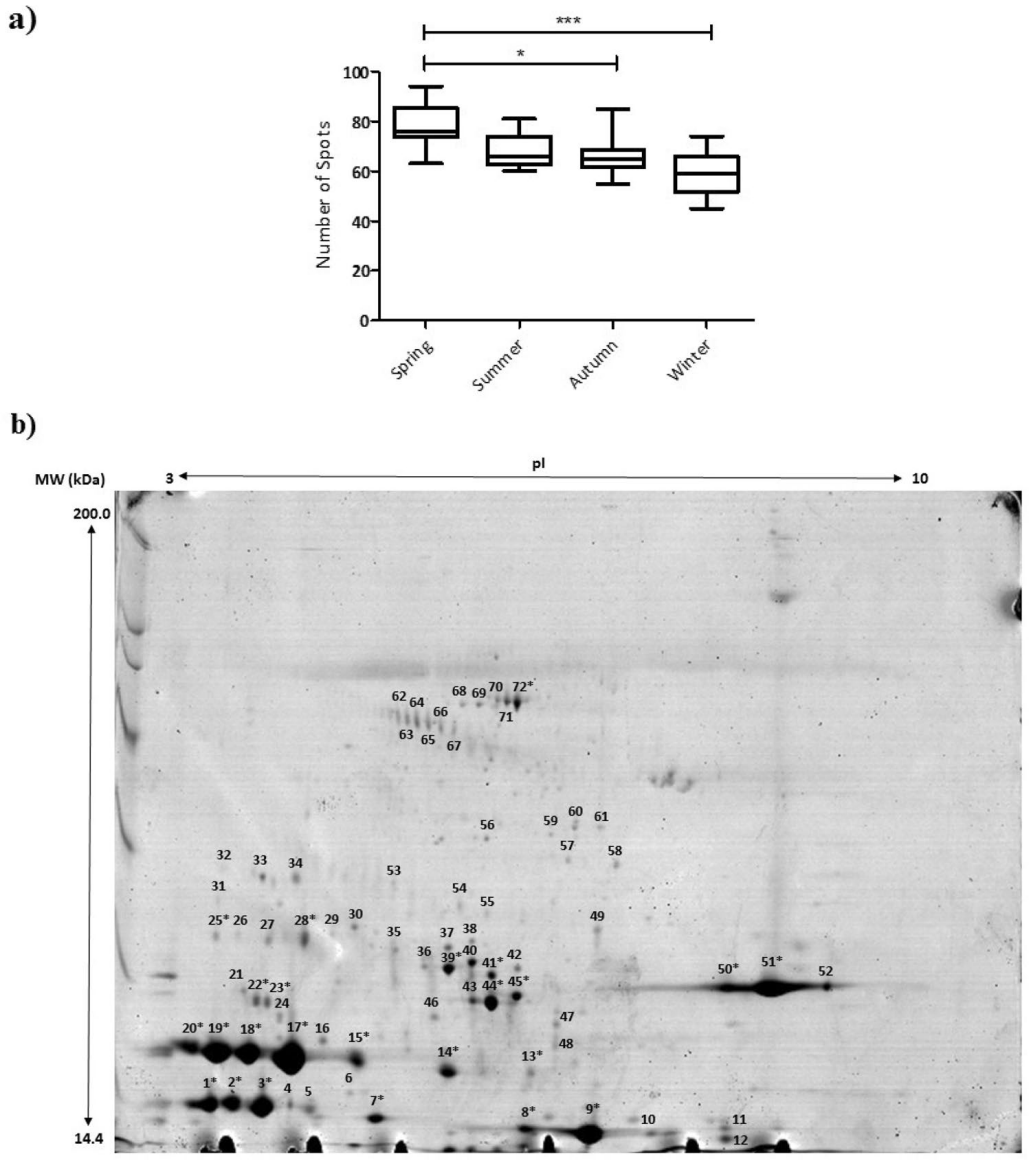
Spots representing proteins from the seminal plasma of stallions in climatological seasons. (**a**) Boxplot of the total number of spots detected by climatological season in gels from the analysis by two-dimensional electrophoresis (2-DE) of the seminal plasma. Three stallions were assessed for different seasons over 1 year, in three replicates. Significant differences in the averages of the number of protein spots by the Tukey test (*p* < 0.05) between spring and autumn gels (*) and spring and winter gels (***) were detected. (**b**) Two-dimensional reference gel for assessment of the protein profile in the seminal plasma of Mangalarga Marchador stallions. The gel contains the 72 spots, on average detected in the seminal plasma. This gel, corresponding to Stallion 1 in the spring season, was used as a reference gel since this treatment showed all the analysed spots among the 2-DE gels in Fig. 2. The proteins were separated using pH 3–10 immobilised pH gradient (IPG) strips (24-cm, linear) and 12.5% T SDS-PAGE gels. Broad-Range Molecular Marker (Bio-Rad) was used (14.4–200 kDa). pI, isoelectric point. MW, molecular weight.

Although the protein profiles were not identical, the changes in the proteome were similar among the animals throughout the climatic seasons, as well as the total number of detected protein spots (Figs. 2, 3 and Supplementary Fig. 6), The total number of spots detected in the 2-DE gels (Fig. 2 and Supplementary Figs. 3, 4, 5) decreased over the seasons (larger for spring, decreasing in summer, autumn and winter), either for the mean value for the three animals per seasons (Fig. 3a) or for individual values per animal (Supplementary Fig. 6). Boxplot (Fig. 3a) used to visually evaluate median, symmetry, and discrepancies (outliers) showed the highest number of protein spots in spring. As well as the median, the average number of spots was significantly higher (*p* < 0.05) in the spring than in the non-breeding season.

In this work, the gel corresponding to Stallion 1 in spring was used to represent the general protein profile of the breed used as the model (Mangalarga Marchador) (Fig. 3b) since it contained the largest number of spots detected among the treatments. Considering the 72 protein spots detected on average for the 2-DE gels (Table 1), eight different proteins, either as a single spot or as proteoforms, were identified in 24 spots by Mascot Daemon and PEAKS. These proteins were the Horse Seminal Plasma proteins, HSP-1 (12 spots) and HSP-2 (1 spot), neutrophil gelatinase-associated lipocalin—isoform X1 (1 spot), kallikrein (4 spots), major allergen Equ c 1-like (2 spots), BPI fold-containing family A member 1 (1 spot), CRISP-3 (2 spots) and BSA (1 spot).

**Table 1:** Proteins identified in the seminal plasma of Mangalarga Marchador stallions throughout the climatic seasons. The proteins were separated using two-dimensional electrophoresis, analysed by MALDI-TOF/TOF equipment, identified by the MASCOT software and PEAKS algorithm and validated by the SCAFFOLD software against the NCBInr, NCBI RefSeq, *Equidae* (from NCBInr) and UniProtKB/Swiss-Prot databases in *Equus caballus*.

Protein name	Spot ID(***)	pI Exp/pI Theo	MW Exp/MW Theo	Access number (in database)	Database	Mascot score	Sequence coverage (%)	Prob. ^(1)^	NPIV ^(3)^	Peptide sequence	Prob. ^(2)^
HSP-1 (Horse Seminal Plasma protein 1)	1	3.32/7.91	20,509/ 19,871	XP_001501767 gi|194,215,547	NCBI RefSeq NCBInr	171	22	100%	03	(R)GKWYFDCTR(A) (K)WYFDCTR(A) (K)CVFPFVYR(G)	90% 95% 100%
	2	3.47/7.91	20,801/ 19,871	XP_001501767 gi|194,215,547	NCBI RefSeq NCBInr	125	10	100%	03	(R)GKWYFDCTR(A) (K)WYFDCTR(A) (K)CVFPFVYR(G)	99% 99% 100%
	3	3.69/7.91	19,937/ 19,871	XP_001501767 gi|194,215,547	NCBI RefSeq NCBInr	143	15	100%	02	(K)WYFDCTR(A) (K)CVFPFVYR(G)	97% 99%
	7	4.52/7.91	18,057/ 19,871	XP_001501767 gi|194,215,547	NCBI RefSeq NCBInr	72	14	-	-	(K)CAFPFNYR(G) (K)WYFDCTR(A) (K)CVFPFVYR(G)	-
	9	6.88/7.51	15,455/ 18,917	XP_001501767 gi|194,215,547	NCBI RefSeq NCBInr	72	8	97%	01	(K)CFFPFVYR(G)	92%
	14	5.53/8.15	25,753/ 14,352	gi|1,168,021 SP1_HORSE	NCBInr Swiss-Prot	47	6	93%	01	(K)CAFPFVYR(G)	93%
	15	4.77/8.15	26,267/ 14,487	gi|1,168,021 SP1_HORSE	NCBInr Swiss-Prot	80	13	99%	01	(K)CVFPFNYR(G)	95%
	17	4.25/8.15	26,267/ 14,487	gi|1,168,021 SP1_HORSE	NCBInr Swiss-Prot	91	13	99%	02	(K)CVFPFNYR(G) (K)CAFPFVYR(G) (R)TDSFYR(W)*	91% 93%
	18	3.82/8.15	26,528/14,487	gi|1,168,021 SP1_HORSE	NCBInr Swiss-Prot	153	23	100%	02	(K)CVFPFNYR(G) (K)CAFPFVYR(G)	94% 90%
	19	3.51/8.15	26,594/14,487	gi|1,168,021 SP1_HORSE	NCBInr Swiss-Prot	114	18	100%	02	(K)CVFPFNYR(G) (K)CAFPFVYR(G)	93% 90%
	20	3.29/8.15	26,858/14,487	gi|1,168,021 SP1_HORSE	NCBInr Swiss-Prot	153	23	100%	02	(K)CVFPFNYR(G) (K)CAFPFVYR(G) (R)YYDCTR(T)*	99% 99%
	25	3.44/7.91	33,841/19,871	XP_001501767 gi|194,215,547	NCBI RefSeq NCBInr	77	14	-	-	(K)CAFPFNYR(G) (K)WYFDCTR(A) (K)CVFPFVYR(G)	-
HSP-2 (Horse Seminal Plasma protein 2)	8	6.28/7.53	15,675/16,031	gi|40,644,080 CAE46516	NCBInr GenBank	40	7	-	-	(K)CFFPFVYR(G) (Q)YYDCTR(T)*	-
Lipocalin 2 or Neutrophil gelatinase-associated lipocalin isoform X1	13	6.80/5.95	25,499/23,247	XP_001501198 gi|338,720,560	NCBI RefSeq NCBInr	51	5	95%	01	(R)YFGVQSYIVR(V)	95%
Major allergen Equ c 1	22	3.89/4.59	28,011/21,629	XP_001489373 gi|149,738,827	NCBI RefSeq NCBInr	92	10	99%	01	(K)DRPFQLLEFYAR(E) (K)YDGYNVLR(I)*	97%
	23	4.07/4.59	27,804/21,629	XP_001489373 gi|149,738,827	NCBI RefSeq NCBInr	148	15	100%	02	(K)YDGYNVLR(I) (R)EPDVSPEIKEEFVK(I) (R)VFVDLIR(A)**	97% 98%
BPI fold-containing family A member 1-like	28	4.49/6.28	32,248/24,465	XP_005604802 gi|545,200,759	NCBI RefSeq NCBInr	44	4	92%	01	(R)WVPVPFR(C)	92%
Kallikrein-1E2	39	5.47/5.44	30,393/21,927	gi|18,250,639 CAD20985	NCBInr GenBank	103	16	99%	02	(K)YLRPYDDISHDLMLLR(L) (R)GSGTLQCVELR(L)	99%
	41	6.43/5.44	29,871/21,927	gi|18,250,639 KLK2_HORSE CAD20985	NCBInr GenBank	74	8	95%	01	(K)YLRPYDDISHDLMLLR(L)	99%
	44	6.42/5.44	28,713/21,927	gi|18,250,639 KLK2_HORSE CAD20985	NCBInr Swiss-Prot GenBank	102	9	95%	01	(K)LGSTCYTSGWGLISTFTNR(G)	100%
	45	6.69/5.44	29,142/21,927	gi|18,250,639 CAD20985	NCBInr GenBank	59	9	95%	01	(K)YLRPYDDISHDLMLLR(L)	98%
HSP-3/CRISP3 (Cysteine-Rich Secretory protein 3)	50	8.81/7.42	30,019/28,202	gi|3,023,562 CRIS3_HORSE	NCBInr Swiss-Prot	95	7	95%	01	(K)TPNAVVGHYTQVVWYSSYR(V)	100%
	51	9.12/7.42	30,019/ 28,202	gi|3,023,562 CRIS3_HORSE	NCBInr Swiss-Prot	139	7	95%	01	(K)TPNAVVGHYTQVVWYSSYR(V)	100%
Albumin	68 to 72	6.08/ 5.89	80,012/ 70,490	gi|76,363,596 Q5XLE4	NCBInr Swiss-Prot	155	5	100%	02	(R)RHPYFYGPELLFHAEEYK(A) (K)DVFLGTFLYEYSR(R)	97% 100%

*Peptide identified only by PEAKS.

**Peptide identified by de novo sequencing. ***Number of the protein spots on the reference gel in Fig. 3b. ^(1)^Probability of identification for protein (Scaffold). ^(2)^Probability of identification for peptide (Scaffold). ^(3)^NPIV—number of peptides identified and validated.

**Figure 4:**
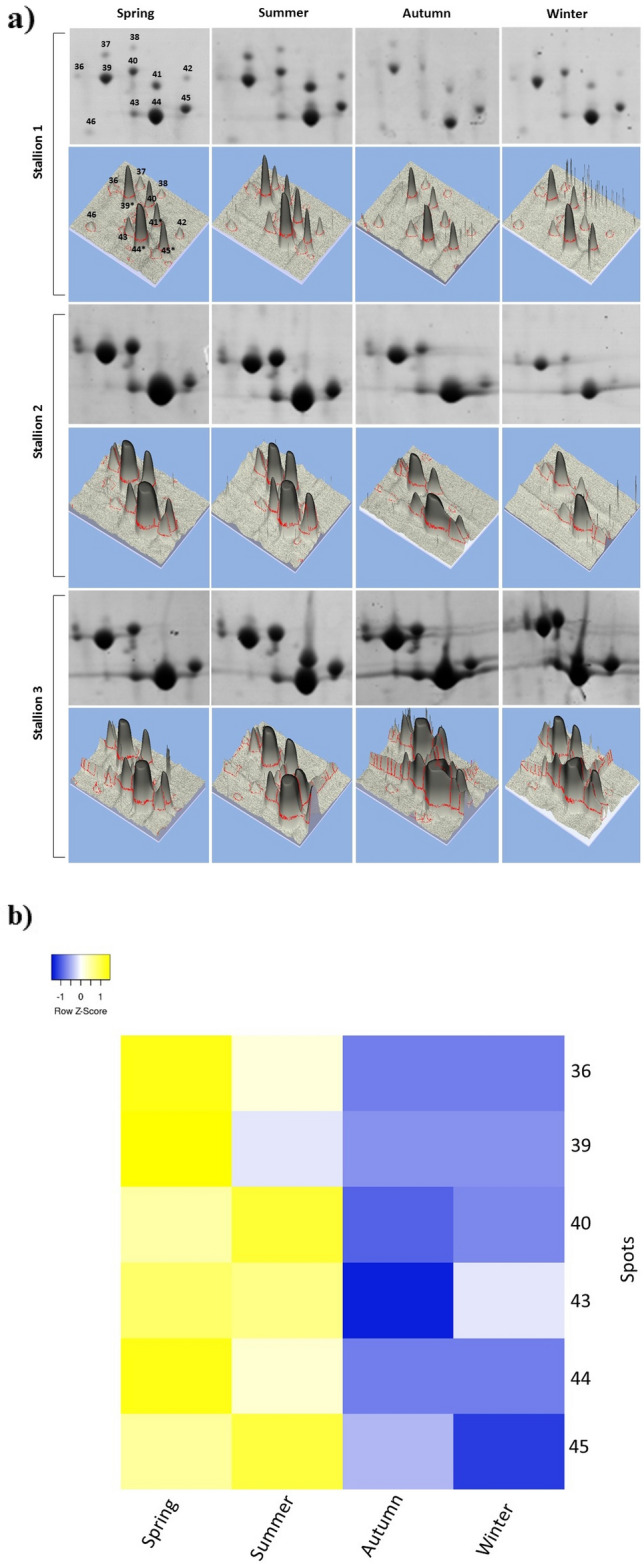
Intensity of kallikrein proteoforms in different climatic seasons. (**a**) Spot region in kallikrein-enriched two-dimensional gels (Spots 36–46) and the corresponding three-dimensional association. The images of the complete gels from which the spots were cut and are presented in the supplementary information file, highlighted in the red frames. (**b**) Heatmap of spots in the region enriched with kallikreins present in the two-dimensional gels of the three stallions. Yellow blocks indicate high protein abundance and blue blocks low protein abundance. The numbers of the points relative to Fig. 3b are on the right side of the heatmap. In (**a**) the ImageMaster 2D Platinum 7.0 software (GE Healthcare, https://imagemaster-2d-platinum.software.informer.com/7.0/) was used to create the 2D and 3D images by selecting the kallikrein-enriched region; figures were edited manually, obeying the size proportion for each treatment. In (**b**) the online Heatmapper software (http://heatmapper.ca/expression/) was used, in which the mean %vol data of the spots identified as kallikreins were submitted only for Animals 1 and 2 (for the spots common to both animals); the data were normalised before being sent to the software, taking into account the spots corresponding to the spring season as a standard.

Assessing proteoforms, HSP-1 was identified in spots 1, 2, 3, 7, 9, 14, 15, 17, 18, 19, 20 and 25 and HSP-2 in spot 8 (Fig. 3b). There were no differences (*p* > 0.05) in the relative abundances of HSP-1 and HSP-2 over the seasons. By analysis of the tryptic peptides (Table 1) recovered from the mass spectra (MS) of the 13 HSP spots (Fig. 3b), six HSP-1 and two HSP-2 proteoforms were identified (Fig. 4) by computational tools for sequence similarity (Blastp—NCBInr) and multiple sequence alignment (Clustal Omega). Two HSP-1 proteoforms (Supplementary Fig. 7a) were identified for spots 1, 2, 3, 7 and 25 (by the sequences (K)WYFDCTR(A) and (K)CVFPFVYR(G) in Table 1), three proteoforms (Supplementary Fig. 7b) for spots 15, 17, 18, 19 and 20 (by the sequence (K)CVFPFNYR(G) in Table 1) and a unique proteoform for spot 9 (by the sequence (K)CFFPFVYR(G) in Table 1), resulting in six HSP-1 proteoforms for 12 spots. Two HSP-2 proteoforms (Supplementary Fig. 7c) were identified for spot 8 (by the sequences (K)CFFPFVYR(G) and (Q)YYDCTR(T) in Table 1). The phylogram (Clustal Omega) representative of all these protein sequences showed that the HSP-1-like proteoform (spot 9) was closer to the HSP-2 (spot 8) than the other HSP-1 proteoforms (Supplementary Fig. 7d). Analysis of the number of copies in the reference genome (tBlastn) indicated a unique access code representing all proteins identified as HSPs (HSP-1 and HSP-2) and an additional access code associated with the HSP-2 protein.

The CRISP-3 (HSP-3), in spots 50 and 51, presented similar abundances (p > 0.05) throughout the climatic seasons. Tools for sequence similarity by NCBInr and multiple sequence alignment by Clustal Omega allowed to recover five protein sequences (Supplementary Fig. 8a, b) representative of these spots, in two similar groups. The phylogram (Clustal Omega) representing these protein sequences corroborated the generation of the two groups obtained by the sequence alignments (Supplementary Fig. 8c). Analysis in the reference genome (NCBIrefseq_genomes) recovered a unique entry corresponding to the five protein accessions identified, evidencing that different CRISP-3 proteoforms are important for the reproductive events.

Four proteins detected with similar abundances in the seminal plasma throughout the climatic seasons (Fig. 2) were considered natural controls in this work. They were (spots in Fig. 3b) serum albumin proteoforms (ALBU_HORSE, spots 68–72), major allergen Equ c 1-like (ALL1_HORSE, spots 22–23), neutrophil gelatinase-associated lipocalin isoform X1 or lipocalin-2 (F6TIR2_HORSE, spot 13) and BPI (bactericidal permeability-increasing protein) fold-containing family A member 1-like (F7AX88_HORSE, spot 28).

Unlike the other proteins, Kallikrein-1E2 (KLK2_HORSE) showed a differential abundance (*p* < 0.05) in the seminal plasma of stallions throughout the year (Figs. 3b, 4, Table 1). The KLK2 was validated by scaffold in four spots (39, 41, 44 and 45), and seven more spots were confirmed by manual comparison of the mass values in the spectra of the tryptic peptides (Supplementary Fig. 9 and Table 2), comprising the spots 36 to 46 in Fig. 3b. By using computational tools, the tryptic peptides of the KLK proteoforms were assessed for sequence similarity by NCBInr (Blastp) and multiple sequence alignment by Clustal Omega. Three representative KLK sequences were recovered from spots 39, 41, 44 and 45 (Supplementary Fig. 10a), which were used for in silico analyses. The phylogram (Clustal Omega) indicated two groups (Supplementary Fig. 10b). Analysis of copy numbers for the genes (NCBIrefseq_genomes) recovered nine accesses, described on nine different chromosomes, indicating the occurrence of proteoforms and evidencing, once again, the need for extensive studies related to the genetic and post-translational diversity and the involvement of KLK proteoforms of the seminal plasma of stallions to understand the reproductive seasonality.

**Table 2:** Ions with isotopic patterns in the mass spectrometry profile of the 11 spots identified as kallikreins in the seminal plasma of stallions throughout the climatic seasons. Four spots were identified as Kallikrein-1E2 and validated by Scaffold (39, 41, 44 and 45), and another seven spots were identified by comparing the general protein profiles obtained (peak list) with the mass values of the tryptic peptides that were recovered from theoretical cleavage of Kallikrein-1E2 by trypsin, using the PeptideMass software.

Ions with isotopic patterns in MS profile (Da)	Spots										
	36	37	38	39	40	41	42	43	44	45	46
**645.754**	X	X	–	X	X	X	X	X	X	X	X
**2020.334**	–	–	–	X	X	X	–	X	X	X	–
1218.4853 (1162.326 + 57.05)**	X	–	–	X*	X	X	–	X	X	X	–
**1364.598 (1307.548 + 57.05)**	X	X	X	X	X	X	X	X	X	X	X
**2035.0195 (2020.334)**	X	X	X	X*	X	X*	X	X	X	X*	
**2121.4048 (2064.300 + 57.05)**	X	X	X	X	X	X	X	X	X*	X	–
**2717.235 (2660.185 + 57.05)**	X	X	X	X	X	X	X	X	X	X	X
**1091.05 (1034.195 + 57.05)**	–	X	X	–	X	X	X	–	–	–	–

*Represents the peptide sequence recovered from MS analysis.

**The value 57.05 Da was added to the peptides when alkylation by acetamide occurred.

### Seasonality in kallikrein proteoforms

By assessing 2-DE gels, KLKs were identified by the MASCOT software and PEAKS algorithm in the spots with the highest intensities (spots 39, 41, 44 and 45 in Fig. 3b). The other spots in the region enriched in KLKs (spots 36 to 46) were identified by comparing the ion profile and the mass values of the ions in the spectra (MALDI-TOF/MS1) of the tryptic peptides (Supplementary Fig. 9 and Table 2). The 11 spots (spots 36 to 46) corresponding to KLK proteoforms, in combination with the nine gene accessions that had been observed for KLKs from the sequences of the identified tryptic peptides, corroborate the wide participation of different KLK proteoforms in the circannual control for horse reproduction.

## Discussion

The BGTHI values and the luminous intensity in the stalls and paddocks are in agreement with Hahn and Mader^21^, who reported that similar BGTHI values (72 to 78) are ideal for long-day animals. The cortisol concentrations in the bloodstream of the stallions are lower than the mean value of 5.12 μg dL^−1^ found by Hoffsiset et al.^22^ in plasma of healthy adult horses in non-stressful conditions.

Regarding the similar total sperm count for all seasons, given the higher semen and lower sperm concentration per mL, our results corroborate the findings of Waddington et al.^23^ and Picket et al.^24^, who reported that seasonality affects stallion seminal plasma production more significantly than sperm production. Although there were differences in semen physical and morphological parameters among seasons, the seasonal average values obtained do not compromise the fertility of the stallions, making all of them suitable for reproduction throughout the year. This fact might look contradictory, but such behaviour favours production and semen cryopreservation in the colder seasons (between the breeding seasons). Moreover, in the colder seasons, stallions produce higher sperm concentrations and smaller seminal volumes, leading to a higher number of frozen semen doses.

The similarity in testosterone blood plasma concentration throughout the year suggested the non-occurrence of reproductive disorders since this hormone is responsible for the development of accessory sex organs. Testosterone is also involved in epididymal epithelium functionality, impacting sperm maturation and the production of seminal plasma proteins^24^.

When submitted to anaerobic conditions, similar to those in the uterine environment, the semen with a higher concentration of endogenous glucose had greater resistance to membrane disruption; sugar molecules have the ability to interact with the membrane lipid bilayer, reorganising and stabilising it^12^. It is worth mentioning that the energy obtained per glucose molecule is substantially reduced in anaerobic environments. Also, glucose molecules, as polyhydroxylated compounds, play important roles as osmotic agents, protecting the structure of functional proteins in solution.

Putting together the results obtained in this work, it is worth mentioning that the amount of sperm produced throughout the year is similar; however, in warmer seasons, there is a lower concentration of spermatozoa in the ejaculate (Fig. 1a), with a higher percentage of injured sperm (Fig. 1b) in relation to the cold seasons. In warmer seasons, the higher volume of semen, which contains greater glucose and cholesterol concentrations, shows that the liquid fraction of the ejaculate can act as an important positive factor for the occurrence of seasonality. In combination, high levels of glucose, cholesterol and seminal plasma volume in the warm seasons may indicate adjustments of the reproductive glandular system to the breeding season in horses. Thus, disentangling the seminal net fraction, including the differential proteome profile throughout the year, helps to understand equine breeding seasonality.

The 2-DE procedure was chosen to assess the differential seminal plasma proteomes in this work as we are interested in the quantitative identification of proteoforms for the major equine seminal plasma, such as BSP 1 (also termed Horse Seminal Plasma Protein (HSP)-1), CRISP-3 and Kallikrein-1E2^25^. Similar, but not identical gels, evidenced the influence of seasonality on the protein profile of the stallions and, consequently, on the characteristics of their seminal plasma, as also reported by Novak et al.^26^. The highest number of protein spots in spring, decreasing towards summer, autumn and winter, reveals the occurrence of different types of proteins (spots) in the breeding season (spring–summer), although the concentrations of total protein were similar throughout the year evaluated. The variation of the proteoforms as a function of the reproductive seasonality can explain this event in horses. The asymmetric position of the second quartile (median) in the boxes for Boxplot tool, used to visually evaluate median, symmetry and discrepancies for the breeding season (spring–summer), indicated greater variability of the animals in terms of the different types of proteins in the seminal plasma. The absence of outliers in all treatments stands out.

The HSP-1 and HSP-2, members of the Binder of Sperm Proteins (BSP)^25^, comprise 70%–80% of the protein concentration in the seminal plasma^16^, making up the most abundant group. Despite the large number of HSP-1 and HSP-2 spots detected, similar to that reported by Garcia et al.^27^, there were no differences (*p* > 0.05) detected in their relative abundances over the seasons, although the analysis of the number of copies in the reference genome indicated a unique access code representing HSPs. These results point to the importance of studying HSP proteoforms in equine seminal plasma to determine their participation in breeding events. The HSP-1 and HSP-2 are homologous to BSP1 and BSP2 from bovines; BSP1 are described as multifunctional, polydisperse, multimeric self-associated protein molecules^25^. In the current work, stallions had similar reproductive profiles, and thus, no differences in the abundance of HSPs throughout the year were detected. As already reported for seasonal animals, the seminal plasma-binding proteins show variability for animals with different reproductive capacities^28^.

The CRISP-3 proteoforms are in high concentrations in the seminal plasma of stallions when compared to other domestic animals^29,30^. High abundances of CRISP-3 are associated with increased fertility rates^26^, probably through the spermatozoa protection of polymorphonuclear neutrophils (PMNs) from the uterine lumen^31,32^. Also, for CRISP-3, the similar abundances in the 2-DE profiles along the climatic seasons seem to be the standard since all the stallions evaluated were efficient sires.

Serum albumin proteoforms (ALBU_HORSE) are produced by the liver, transporting molecules in the blood such as steroids, fatty acids and hormones; their proposed function is the protection of the plasma membrane and the maintenance of sperm motility by absorbing lipid peroxides that damage cells^33^. The major allergen Equ c 1-like (ALL1_HORSE) belongs to the lipocalin family, one of the top five main purified horse allergens^34^; it acts by carrying small hydrophobic molecules such as odorants, steroids and pheromones^35^, being most probably a seminal plasma contaminant^36^. Neutrophil gelatinase-associated lipocalin isoform X1 or lipocalin-2 (F6TIR2_HORSE) is involved in innate immunity to bacterial infection^37^. Also, BPI (bactericidal permeability-increasing protein) fold-containing family A member 1-like (F7AX88_HORSE) is structurally related to proteins capable of binding lipophilic substrates, phospholipids and lipopolysaccharides, being therefore directed against Gram-negative bacteria^38^. Both lipocalin-2 and BPI may play protective roles in fertilisation.

Kallikreins are regulatory serine proteases^39^ whose expression related to animal reproduction is controlled by androgens. The KLKs are synthesised as inactive proteins^40^, and the kininogen of the seminal plasma is their specific enzymatic substrate^41^. Both kallikrein-kinin system (KKS) and renin-angiotensin system (RAS) are key proteolytic systems that control a wide spectrum of systemic and local physiological activities, including animal reproduction. The KKS and RAS act in opposite ways since the activation of one system counteracts the metabolic effects of the other. Both systems are controlled by the angiotensin-converting enzyme (ACE). Renin cleaves the N-terminal portion of angiotensinogen to form the biologically inactive decapeptide angiotensin I, which is then hydrolysed by ACE to form the octapeptide angiotensin II (Ang II), biologically active as a vasoconstrictor^42^. The ACE also inhibits the bradykinin synthesis, a vasodilator^43^, inhibiting the kallikrein-kinin system (KKS). Then, an increase in KLK abundance in the warm seasons, as reported here, can be related to the lower ACE activity. In the breeding season, a lower sperm motility was observed, probably due to the reduced production of AngII, in turn caused by the lower ACE activity and the consequently higher KLK abundance.

The environmental temperature is one of the regulatory factors of the KKS and RAS pathways. In a systematic review and a meta-analysis, respectively, with 23 and 14 studies, Wang et al.^44^ observed a consistent, statistically significant, inverse association between temperature and blood pressure. In warmer seasons, blood pressure will be reduced due to ACE inhibition, which agrees with Borghi and Omboni^42^, who reported the ACE inhibitors as antihypertensive agents. This means that, in hot seasons, when the inhibition of the renin-angiotensin system (RAS) occurs, the kallikrein-kinin system (KKS) will be activated.

In addition, Vinson et al.^45^ reported that AngII, produced by ACE activity, interacts with spermatozoa, triggering events favourable to sperm motility in rats and humans. In the breeding season (spring–summer), higher KLK abundances are in agreement with inhibition of the RAS system (lower ACE activity and lower AngII production), although the lower mean values of the characteristics observed in the present study did not compromise seminal parameters of fertile and sexually active animals.

Several studies have shown the presence of KLKs in the seminal plasma, but their function in reproductive seasonality has not been fully elucidated. Evaluating proteins in the seminal plasma of domestic animals, Druart et al.^30^ identified three KLK bands in a denaturing gel for stallions but not for boar, bull, buck, ram, alpaca, and camel. Jobim et al.^20^ have detected KLKs in the semen of stallions, considering them as detrimental to cryopreservation. Recent reports about KLK proteoforms in seminal plasma were not able to reveal their functions in reproduction. Höfner et al.^25^ reported KLK-E1 as one of the major quantitative equine proteins in seminal plasma, besides HSP-1 and CRISP-3, similarly to Guasti et al.^18^, whose also reported 13 spots in 2-DE gel as KLK proteoforms. Kareskoski et al.^46^ reported two albumin and two kallikrein proteoforms related to sperm motility. The abundances of KLKs in the gels for the different climatic seasons were compared by heatmaps, showing that the different proteoforms are, on average, more abundant in the breeding season: spring–summer. We can infer, according to our results, that KLKs are then directly related to the reproductive seasonality of stallions.

The KLK proteoforms must be individually studied in the breeding and non-breeding seasons specifically for stallions since Druart et al.^30^ identified KLKs in stallion seminal plasma but not in the plasma of other domestic animals. The variability in the proteinase activity by KLK proteoforms seems to be an important event in the breeding season and essential to our understanding of the correlation of different KLK proteoforms in seasonal breeding.

The information obtained in this work about the influence of bioclimatic aspects on the morphological and physical characteristics of semen and the differential protein abundances are in accordance with the characteristics of horse breeding, which is seasonal polyoestrous, with two reproductive periods established throughout the annual climatic seasons, the natural breeding season (spring–summer) and the non-breeding season (autumn–winter). Regarding the quantitative characteristics studied in this work, there was a greater abundance of KLK proteoforms under the conditions of increased day light and temperature, which is the breeding season (spring–summer), as well as a greater semen volume, higher glucose and cholesterol concentrations and a higher total number of protein spots in 2-DE gels when compared to the non-breeding season (autumn–winter).

The physical characteristics of semen that were not influenced by the climatic seasons in the current work were sperm vigour, total number of ejaculated sperm and percentage of defects (major, minor or total). There were also no differences in the concentrations of total soluble proteins in the seminal plasma and blood cortisol between the climatic seasons nor in the abundances of HSP-1, HSP-2 and CRISP-3 (HSP-3) proteoforms, which can be explained by the high reproductive capacity of the animals studied.

Finally, this work showed that, for fertile stallions, KLKs present greater abundances in the breeding season than in the non-breeding season. This difference did not occur with HSP and CRISP-3 proteins, although these three classes are described as the main proteins present in seminal plasma in quantitative terms. Then, only the KLKs differed over the circannual rhythm of stallion reproductive activity (Table 1). Both the number of KLK spots (reporting different types) and the intensity of the spots (different abundances) on the gels differed.

By 2-DE analysis, this work identified 11 KLK proteoforms in the seminal plasma of stallions collected throughout the year, with high significance in identification. Nine representative accessions of KLKs were found by analysing the number of gene copies for *Equus caballus* based on the protein sequences identified from seminal plasma. These genes/proteins may be involved in different regulatory processes^39^. The KLKs act in response to the presence of steroids and other hormones, although the mechanisms involved in the expression of these proteinases are not fully understood.

In this work, for fertile stallions, the morphological and physical–chemical factors suggest that the KLK proteoforms are conditioned by the climate or climatic season. In the breeding season (spring–summer), the highest abundances of KLKs in seminal plasma were accompanied by greater volumes of semen, higher concentrations of glucose and cholesterol and a higher number of protein spots in 2-DE gels (including KLKs), besides low sperm motility, which may be associated with the inhibition of the RAS system and might be conditioned by climate. Also, in the non-breeding season (autumn–winter), sperm were produced in numbers similar to those in the breeding season, with similar levels of vigour and defects, but with higher motility and higher relative concentration in seminal plasma, allowing inferences about the advantages in collecting stallion semen in the non-breeding season for freezing and commercialisation. This suggestion is in line with a previous study, which reported that KLKs can negatively interfere with the freezability of stallion semen^20^.

The KLKs showed a direct relationship with the breeding season and, therefore, with the circannual rhythm of reproduction in horses. Unravelling the involvement of KLK proteoforms in reproductive seasonality is a priority for comprehensive studies about the stallion circannual reproductive rhythm and of the influences and consequences of possible bioclimatic and environmental changes. It is worth mentioning that despite the use of Mangalarga Marchador stallions as a model in the current study, our team precludes that the findings described here are applicable to almost all horse breeds since reproductive seasonality is a characteristic of equine species. In this way, other teams are welcome to validate the current findings for different countries and breeds.

## Methods

### Ethics

This study was approved by the Ethics Committee for Use of Animals (CEUA) to be developed with three animals, Process n^o.^ 40/2014, at the Universidade Federal de Viçosa (UFV), City of Viçosa, State of Minas Gerais, Brazil. All methods were carried out in accordance with relevant guidelines and regulations, and all methods are reported in accordance with the ARRIVE guidelines (https://arriveguidelines.org).

### Location and management of animals

The experiment was carried out at the Equideoculture Farm at UFV, City of Viçosa, State of Minas Gerais, Brazil, at an elevation of 649 m and the geographical coordinates of 20°45′14'' South latitude and the meridian of 42° 52′ 54″ West longitude Gr. The climate is Cwa (mesothermal), according to the Köppen classification, with two well-defined seasons, with warm, humid summers and cold, dry winters. Three Mangalarga Marchador stallions (Animal 1, 15 years old and 480 kg; Animal 2, 10 years old and 405 kg and Animal 3, 15 years old and 410 kg) were used as semen donors. During the trial, animals were housed in individual stalls of 16 m^2^ with beds of fat grass changed daily. From 7:00 a.m. to 2:00 p.m., they were released on individual paddies of coastcross (*Cynodon dactylon* (L.) Pers) and Tifton 85 (*Cynodon* spp.) grass of 225 m^2^. The supplementation was composed of concentrated feed consisting of 14% w/w of total protein offered at 1.0% w/w of the body weight of the animal, elaborated according to the recommendations of the National Research Council^47^, elephant grass (*Pennisetum purpureum* cv. Cameroon) with sugarcane (*Saccharum officinarum*) (3.5% w/w + 1.0% w/w of the body weight of the animal, respectively), mineral salt and water ad libitum*.* Prior to the experiment, seminal examination was performed for all three animals, considering them suitable for reproduction according to the standards recommended by the BCAR (Brazilian College of Animal Reproduction)^48^.

### Ambiental parameters

The animals remained in two bioclimatological microenvironments, stalls and paddocks, each containing a maximum-minimum thermometer, a black globe thermometer and a wet bulb and dry bulb thermometer. Light intensity was measured using a luximeter. Information was collected at 7:00 a.m., 9:30 a.m., 2:30 p.m. and 5:00 p.m., once a week. The used environmental parameter was the Black Globe Temperature and Humidity Index (BGTHI)^49^, estimated as BGTHI = 0.72(BGT + WBT) + 40.6, where BGT is the black globe temperature (in °C) and WBT is the wet bulb temperature (in °C).

### Semen collection and seminal plasma analysis

Semen collections were carried out every 2 weeks, over 1 year, totalling 26 collections per animal. Fifteen days before the first collection, the extragonadal reserves were depleted, and a collection was performed daily for 7 consecutive days. The collections were made using an equine artificial vagina (Botucatu model—Biotech Ltda, Botucatu, SP, Brazil) previously filled with water heated to 50 °C and coated with a disposable plastic mucosa lubricated with sterilised Vaseline. In all samples, a mare was used in natural or induced oestrus (20 mg of oestradiol cypionate, ECP—Pfizer Saúde Animal, São Paulo, SP, Brazil), duly contained, to protect the stallions. After collection, the semen volume was measured in a warmed recipient, filtered using a nylon membrane to remove the gel and kept in a water bath at 37ºC. The obtained samples were analysed for total motility, vigour, sperm concentration, total spermatozoa per ejaculate, sperm morphology and plasma membrane integrity, along with the supravital (eosin-blackout) and hypoosmotic test.

Total sperm motility (0% to 100%) and sperm vigour (0 to 5) were analysed at 200 × magnification under a phase contrast microscope by the double-blind method. The sperm concentration was determined in a Neubauer chamber using a 1:20 diluted sample in buffered saline formol solution, 5 min after placing an aliquot of each sample in both compartments of the chamber. The total spermatozoa were obtained by multiplying the volume of ejaculate without gel by the spermatic concentration found. To calculate the percentages of spermatozoa with normal and abnormal morphology, sperm were evaluated on wet preparations between slide and cover slip, and a total of 200 spermatozoa per sample were counted under a light microscope with 1000 × magnification in immersion oil. The percentages of major, minor and total defects were determined according to the BCAR^48^.

Plasma membrane integrity was assessed by epifluorescent probes as described by Harrison and Vickers^50^. An aliquot of semen preheated at 37 °C in a water bath was diluted with the fluorescent probe solution (1:20) containing propidium iodide and carboxyfluorescein diacetate. The mixture was incubated for 15 min, and 100 sperm cells were counted and classified as intact or injured under an epifluorescence microscope (Eclipse Model 80i—Nikon Healthcare Business, Tokyo, Japan) at 1000 × magnification, using a triple band pass filter (D/F/R, C58420) featuring the UV-2E/C (excitation at 340–380 nm and emission at 435–485 nm), B-2E/C (excitation at 465–495 nm and emission at 515–555 nm) and G-2E/C (excitation at 540–525 nm and emission at 605–655 nm), where intact sperm were stained in green throughout their extension, injured sperm were stained in red, and semi-damaged cells were red in the acrosomal region and green in the nucleus region. Supravital analysis was performed using the Eosin-Nigrosin staining procedure^51^ by seminal swab, mixing semen with dye reagent; 100 cells were subsequently counted, and the heads of viable spermatozoa remained colourless.

The functional integrity of the plasma membrane was evaluated using hypoosmotic tests. In a 1.5-mL microtube, 800 μL of distilled water was warmed in a water bath up to 37 °C, and 100 μL of the sample to be analysed was incubated at 37 °C for 15 min. The sample was then fixed with 0.5 mL of buffered saline solution for preservation. Analysis was performed under a phase contrast microscope at 1000 × magnification, under immersion oil, by counting 200 cells per ejaculate; those that presented a doubling or spermatic tail coiling were considered reactive to the test. From the final percentage of reactive cells, the percentage of spermatozoa with bent tails found in the morphological test was subtracted, resulting in the percentage of cells with intact plasma membranes. At the end of the physical evaluations of the semen, the ejaculate was centrifuged at 700 g for 20 min at 4 °C to remove spermatozoa, and the supernatant was stored at − 80 °C.

### Seminal plasma total protein

Seminal plasma samples from each stallion (26 ejaculates per animal) were merged into 13 pools, being grouped every two subsequent samples, following the date of the collections; each pool contained 500 μL of plasma from each ejaculate. Pools were centrifuged at 10,000*g* for 10 min at 4 °C, followed by a second centrifugation at 20,100*g* for 30 min at 4 °C for clarification of the samples. The Coomassie Brilliant Blue G-250 (Bradford^52^) and Bicinchoninic acid (BCA^53^) methods were used to determine the soluble proteins. A standard curve was generated with bovine serum albumin (BSA) as standard protein.

### Total cholesterol and glucose concentrations in seminal plasma

Analyses of the total cholesterol and glucose concentrations in the seminal plasma were performed by enzymatic colorimetric assays, using commercial Bioclin kits (Quibasa, Belo Horizonte, MG, Brazil) for cholesterol monoreagent (K083) and for glucose monoreagent (K082), following the manufacturer's recommendations.

### Serum testosterone and cortisol profile

Blood collections from the jugular vein for both profiles were performed over a period of 24 h, at 20-min intervals between collections, once for each climatic season, resulting in 72 samples per animal in each season, totalling 864 samples. The analyses were performed using the commercial Access Testosterone Reagent Kit 2 × 5 in the Access apparatus (Beckman Coulter, Inc., Brea, CA, EUA) by the chemiluminescence immune-enzymatic technique, according to the manufacturer's specifications.

### Two-dimensional gel electrophoresis and analyses of gel images

After clarifying the seminal plasma by centrifugation, new composite samples were formed by combining the samples collected according to each climatic season. To ensure separation between the assessed periods, equal volumes of the collected samples from each season were combined, except from the first and last 2 weeks of the season. The same procedure was used for all climatic seasons and individually for each stallion. For each composite sample, the electrophoresis analyses were developed in three biological replicates and in three technical replicates.

The samples were separated using two-dimensional polyacrylamide gel electrophoresis (2-DE) according to Novak et al.^26^, with modifications. Isoelectric focusing (IEF) was carried out using an Ettan IPGphor 3 IEF System (GE Healthcare, Chicago, IL, USA) according to the manufacturer’s instructions, using pH 3–10 immobilised pH gradient (IPG) strips (24-cm, linear). Each sample was composed of an aliquot of the seminal plasma containing 500 μg of proteins in rehydration buffer (containing 7 mol L^−1^ urea, 2 mol L^−1^ thiourea, 40 mM dithiothreitol (DTT), 0.5% v/v free ampholytes (IPG-Buffer pH 3–10), 2% w/v 3-[(3-Cholamidopropyl) dimethylammonio]-1-propanesulfonate hydrate (CHAPS) and 0.001% w/v bromophenol blue). Samples were brought to 450 µL using the DeStreak rehydration solution (GE Healthcare, Piscataway, NJ, USA), and each sample was loaded in a reswelling tray, overlaid with the IPG strip and allowed to re-hydrate for 12 h. Strips were then subjected to the first separation under 200 V for 10 h, followed by 500 V for 1 h, a graded current of 800 V h^−1^ applied until 1000 V, following 16,500 V h^−1^ applied gradually until 10,000 V and 27,000 V h^−1^ applied abruptly until 10,000 V. Amperage was fixed at 75 μA per strip and temperature at 20 °C.

After IEF, IPG strips were incubated for 15 min in reducing buffer (6 mol L^−1^ urea, 30% v/v glycerol, 2% w/v sodium dodecyl sulfate (SDS), 1% w/v DTT and 75 mmol L^−1^ Tris–HCl, pH 8.8) and re-equilibrated for an additional 15 min in alkylation buffer (similar to reducing buffer, containing 2.5% w/v iodoacetamide instead of DTT). The equilibrated IPG strips were then loaded and fixed with 5% w/v agarose (in SDS-PAGE running buffer) on the top of homogeneous 12.5% T SDS-PAGE in gel. A 5 × 5-mm piece of paper containing 15 μL of the Broad-Range Molecular Weight Marker (cat.#1610317—Bio-Rad, Hercules, CA, USA) was used. The strips containing the reduced and alkylated proteins were subjected to the Ettan DALTsix Electrophoresis System (GE Healthcare Bio-Sciences AB, Cytiva, Uppsala, Sweden) for separation of the proteins by molecular weight at 10ºC to avoid overheating of the system. When the dye had reached the lower bound of the plates, the proteins in the gels were fixed overnight in a solution containing 10% v/v acetic acid and 40% v/v ethanol. The proteins were then stained with colloidal Coomassie Blue^54^.

The gels were washed with distilled water and stored in 5% v/v acetic acid solution. After that, gels were scanned at 300 dpi using an ImageScanner III (GE Healthcare Bio-Sciences AB, Cytiva, Uppsala, Sweden). The files were saved as .mel format, analysed by ImageMaster 2D Platinum 7.0 (GE Healthcare Bio-Sciences AB, Cytiva, Uppsala, Sweden). Gel analysis produced a unique master gel representative for each sample, using a set of the three gels corresponding to the technical replicates. Proteins in key regions of the master gel were chosen as landmarks. Matching of spots was achieved after several rounds of comparisons by automatic detection of the spots in each representative gel. The spot volumes were normalised to the total spot volume of the gel. The percentage volume of each spot was used to estimate the differential abundances of the proteins among the samples.

### In-gel protein digestion and sample clean-up

After gel comparisons by ImageMaster, each interesting protein spot was excised and de-stained by three washes with 400 µL of 50% v/v acetonitrile (ACN) containing 25 mmol L^−1^ ammonium bicarbonate. Gel pieces were then dehydrated by two incubations with 200 µL of absolute ACN for 5 min, using a Savant SpeedVac Vacuum Concentrator (SPD120—Thermo Fisher Scientific Inc., Asheville, NC, USA). Proteins in the dry gel pieces were trypsinised using 13 ηg.spot^−1^ of sequencing-grade modified trypsin (cat.#V5111—PROMEGA, Madson, WI, USA) by 20 h at 37 °C^55^. Peptides were extracted from gel pieces by incubating thrice with 50 µL of 0.1% v/v trifluoroacetic acid (TFA) and 50% v/v ACN in ammonium bicarbonate (50 mmol L^−1^) for 30 min. Supernatants were concentrated to a final volume of 10 μL, using the same vacuum concentrator system. A blank gel piece, without spots, was subjected to the same procedure to be used as the negative control. Disposable ZipTip Pipette Tips Merck Millipore (EMD Millipore Corporation, Billerica, MA, USA) cleaned up the samples.

### Protein identification and proteomics analysis

The mass spectrometric analyses were developed at the Nucleus for Analysis of Biomolecules (www.nubiomol.ufv.br) at Universidade Federal de Viçosa. Data acquisition was performed on a MALDI-TOF/TOF mass spectrometer model Ultraflex III (Bruker Daltonik GmbH, Bremen, Germany), using α-cyano-4-hydroxycinnamic acid (cod.#8201344—Bruker Corporation, Billerica, MA, USA) as the matrix (5 µg mL^−1^ in 50% v/v ACN and 0.1% v/v TFA) in a proportion of 1:3 (sample: matrix), onto an MTP AnchorChip MTP 600/384 TF target (Bruker Daltonik GmbH, Bremen, Germany). The MS spectra were acquired in the positive ion reflector mode, recording ions up to 3000 Da. External calibration was done using the Peptide Calibration Standard II (cat.#8222570—Bruker Corporation, Billerica, MA, USA). The equipment operates under the flexControl 3.3 software (Bruker Daltonik GmbH, Bremen, Germany). Laser energy and number of shots per segment spectrum were adjusted manually for each protein. Interfering peaks from the matrix were removed, which were the gel controls, trypsin and keratin. Peaks with intensity five times higher than background in the 800- to 3000-m/z range were analysed for protein identification, and the most intense ions were subjected to fragmentation. Using the flexAnalysis 3.3 software (Bruker Daltonik GmbH, Bremen, Germany), spectrum processing and database searches were performed automatically with internal calibration, using the trypsin autolysis peaks. Protein identification was performed using the Mascot Daemon, version 2.5.0 (Matrix Science, London, UK), by comparison with the general protein databases NCBInr, UniProtKB and SwissProt and with an Equidae database obtained from NCBInr. The database search parameters included no restrictions on the protein molecular mass, maximum of one trypsin mis-cleavage, non-fixed modifications of methionine (oxidation) and modifications of cysteine (carbamidomethylation). The peptide mass tolerance was 0.1 Da for the precursor ions in MS spectra and 0.5 Da for the fragment ions in MS/MS spectra. Peptides were identified when the scoring value exceeded the identity or extensive homology threshold value calculated by Mascot Daemon for each protein. The spectra were also evaluated by the PEAKS Studio 7.5 software package (Bioinformatics Solutions Inc., Waterloo, ON, Canada) for the analysis of peptide tags by de novo sequencing.

### Statistical analysis

The statistical software System for Statistical Analysis, version 9.1 (SAEG, http://arquivo.ufv.br/saeg/), was used to evaluate the data. All characteristics studied (physical and morphological aspects of the semen, complementary tests, quantification of proteins, cholesterol, glucose, testosterone and cortisol) were analysed by descriptive statistics (mean and standard deviation). For all quantitative characteristics, the data were subjected to the Lilliefors and Barttlet as well as Cochran tests to verify the normality of the data and the homogeneity of the variables, respectively. For positive results, data were analysed by ANOVA. When they were significant by the F test, the means were compared by the Tukey test at 5% of error probability. This analysis was performed both within each season and among seasons. Data that did not meet the ANOVA assumptions were submitted to non-parametric analysis by the Kruskal–Wallis test. The same procedure was used in the analysis of total protein quantification by the two methods employed, but the means were compared by the F (parametric) test or by the Will Coxon test (non-parametric). The behaviour of all quantitative data was studied within and throughout the seasons by means of regression analysis. Pearson's simple correlation was performed among all the studied characteristics.

## Supplementary Information

Supplementary Information.

## Data Availability

All data generated or analysed during this study are included in this published article and its supplementary information files.
