# Supplementary material for: Kallikrein proteoforms and reproductive parameters in stallion are conditioned by climate
Source: Sci Rep. 2022 Nov 4;12:18690. doi: 10.1038/s41598-022-21350-w (PMC9636271; doi:10.1038/s41598-022-21350-w)
Supplement: Supplementary file 1 — Supplementary Information. [file 41598_2022_21350_MOESM1_ESM.pdf]

Supplementary Material

Kallikrein proteoforms and reproductive parameters in stallion are conditioned by Climate

Renato Lima Senra[1,5], Camilo José Ramírez-López[1,2,5], Marcos Jorge Magalhães-Júnior[1,5], João Gabriel da Silva Neves[1,2,5], Edvaldo Barros[4], Bruna Waddington[2], Simone Eliza Facioni Guimarães[3], José Domingos Guimarães[2], Maria Cristina Baracat-Pereira[1,4,*]

[1]Proteomics and Protein Biochemistry Laboratory, Universidade Federal de Viçosa, Brazil.

[2]Animal Reproduction Laboratory, Universidade Federal de Viçosa, Brazil.

[3]LABTEC-Animal Biotechnology Laboratory, Universidade Federal de Viçosa, Brazil.

[4]Nucleus for Analysis of Biomolecules, Universidade Federal de Viçosa, Brazil.

Universidade Federal de Viçosa, 36.570-900 Viçosa-MG, Brazil.

[5]These authors contributed equally: Renato Lima Senra, Camilo José Ramírez-López, Marcos Jorge Magalhães-Júnior and João Gabriel da Silva Neves.

Corresponding author:

Maria Cristina Baracat-Pereira, Laboratório de Proteômica e Bioquímica de Proteínas, Departamento de Bioquímica e Biologia Molecular, Universidade Federal de Viçosa, 36.570-900 Viçosa – MG, Brazil. E-mail: baracat@ufv.br.

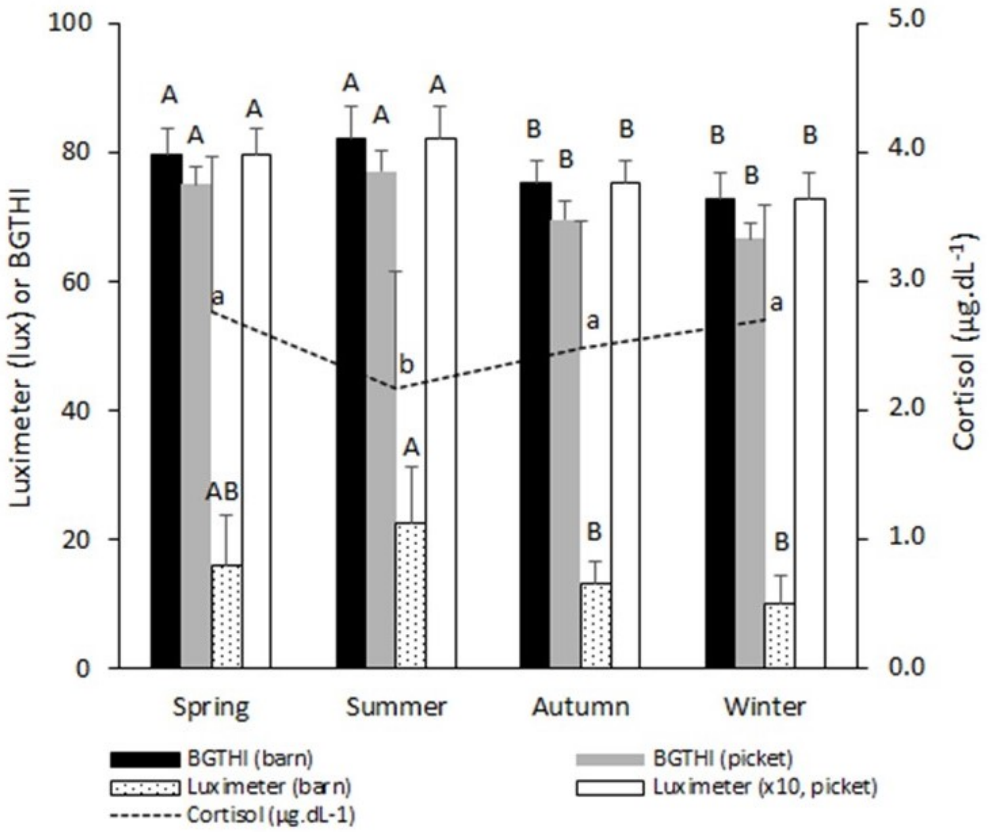

Supplementary Fig. 1 | Climatological parameters and cortisol concentrations in two climatic mini-seasons (barns and pickets). Mean values of the Black Globe Temperature and Humidity Index (BGTHI), luminous intensity and serum cortisol levels were evaluated. Different capital letters in the same bar differ ($p<0.05$) from each other by the Tukey test; different lowercase letters differ ($p<0.05$) from the Kruskal Wallis test.

E		D		C		B		A
0	0.5	1.0	1.5	2.0	2.5	3.0	3.5	4.0
0		25		50		75		100

Statistical analyses: A=4; B=3; C=2; D=1; E=0

Factor	Spring	Summer	Autumn	Winter
BGTHI	4	4	3	3
Luximeter	3.5	4	3	3
Cortisol (μg.dL^{-1})	4	3	4	4
Semen vol. in gel (mL)	3-1=2	4-1 (2.5)	0	3-0 (1.5)
Filtered semen vol. (mL)	3	4	2	2
Motility (score)	2	3-2 (2.5)	4-3 (3.5)	4
Vigor (score)	4	4	4	4
Sptz defects (>s, <s, tt) (nº.)	4	4	4	4
Nº. of sptz in ejaculate	4	4	4	4
Sperm conc. (sptz.mL^{-1})	3	3	4	4
Soluble protein (mg.mL^{-1})	4	4	4	4
Injured sptz (%)	4	4	3	3.5
Semi-injured sptz (%)	4	4	4	4
Intact sptz (%)	3	3	4	3.5
Glucose (mg.dL^{-1})	4	4	3.5	3
Cholesterol (mg.dL^{-1})	3	4	1	2
Spots in gel 2D (median)	72	67	64	58

Supplementary Fig. 2 | Global assessment of the andrological and biochemical factors conditioned by the climate in stallions throughout a year.

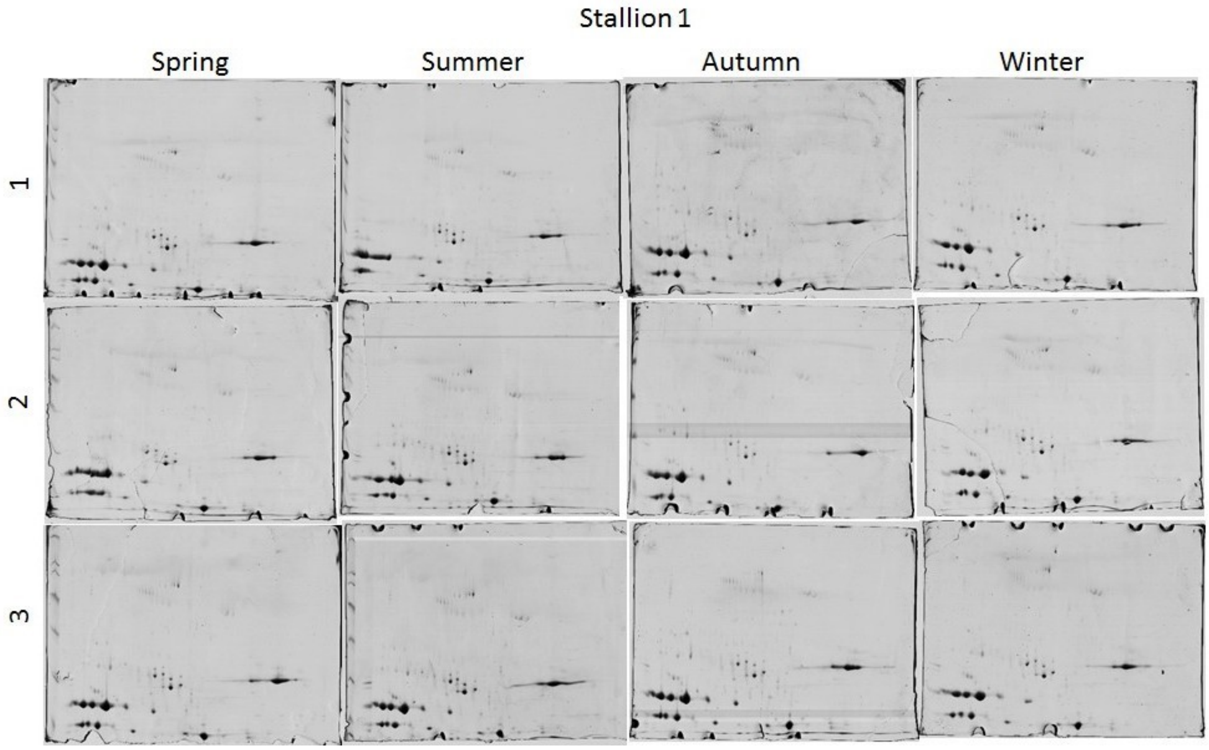

Supplementary Fig. 3 | Protein profile of the seminal plasma of the Stallion 1, throughout a year, in three technical replicates for each treatment. The proteins were separated using pH 3-10 immobilized pH gradient (IPG) strips (24-cm, linear), and 12.5% SDS-PAGE gels. Broad Range Molecular Marker (Bio-Rad) was used (14.4-200 kDa).

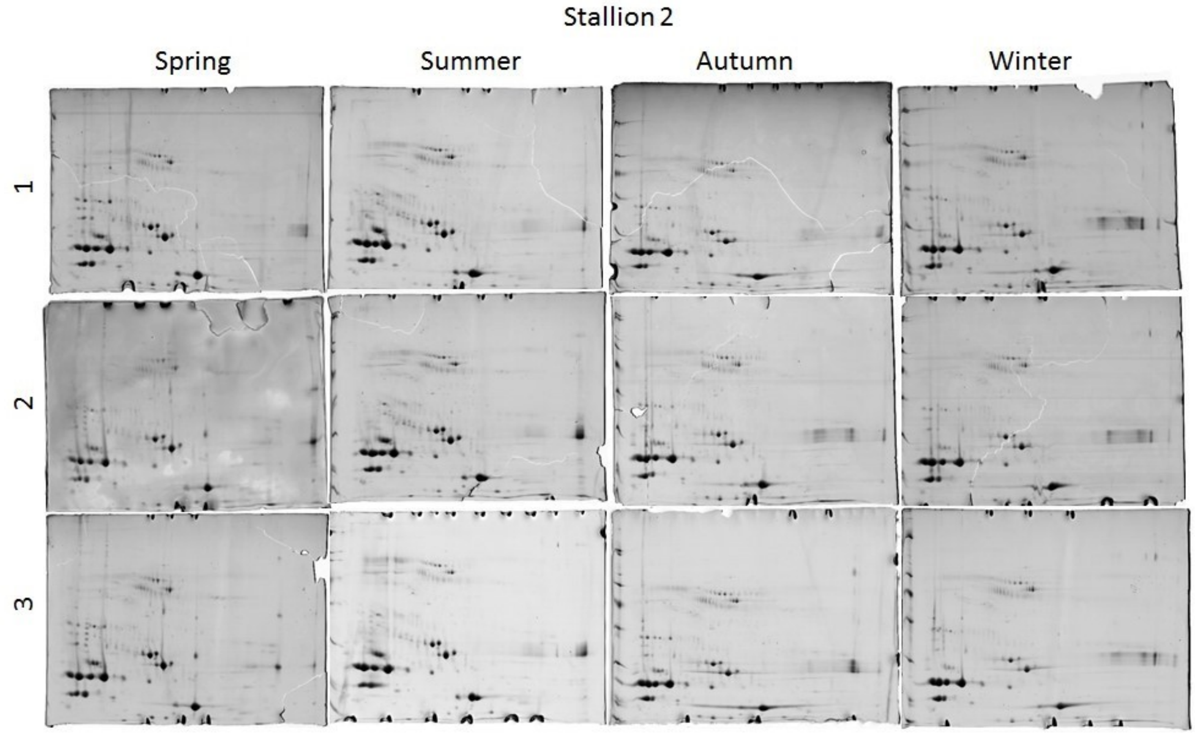

Supplementary Fig. 4 | Protein profile of the seminal plasma of the Stallion 2, throughout a year, in three technical replicates for each treatment. The proteins were separated using pH 3-10 immobilized pH gradient (IPG) strips (24-cm, linear), and 12.5% SDS-PAGE gels. Broad Range Molecular Marker (Bio-Rad) was used (14.4-200 kDa).

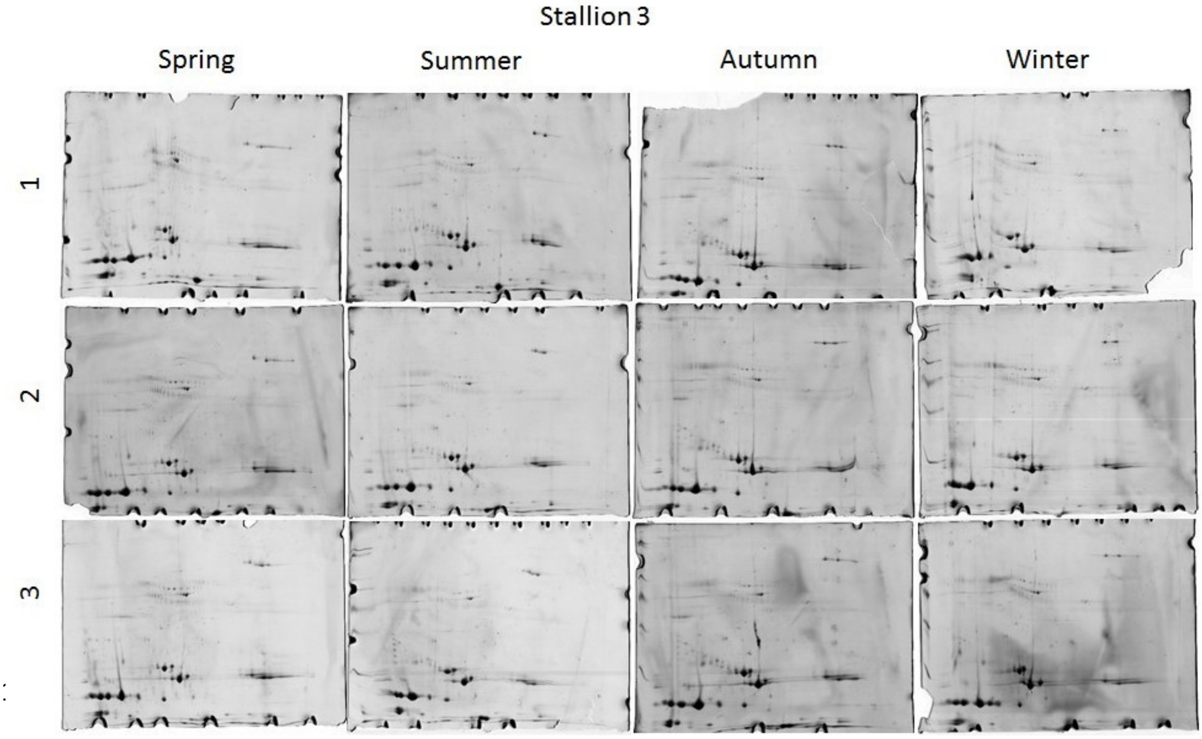

Supplementary Fig. 5 | Protein profile of the seminal plasma of the Stallion 3, throughout a year, in three technical replicates for each treatment. The proteins were separated using pH 3-10 immobilized pH gradient (IPG) strips (24-cm, linear), and 12.5% SDS-PAGE gels. Broad Range Molecular Marker (Bio-Rad) was used (14.4-200 kDa).

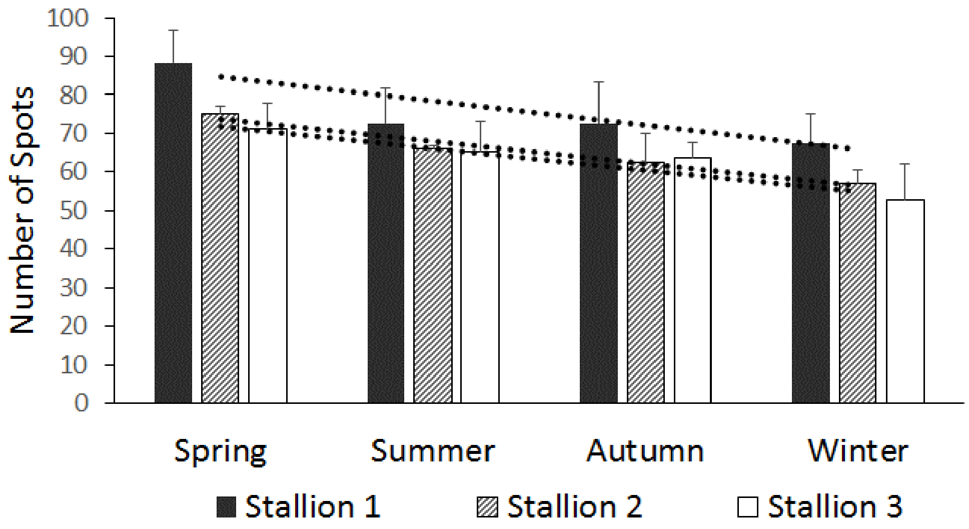

Supplementary Fig. 6 | Number of spots detected for each stallion and climatological season. The number of spots in the two-dimensional gels decreased over the seasons (from spring to summer, autumn and winter) per animal. For the animals, the adjusted lines were: Stallion 1: $y = -6.3\,x + 91.0$, $R^2 = 0.803$; Stallion 2: $y = -5.7\,x + 79.5$, $R^2 = 0.965$; Stallion 3: $y = -5.7\,x + 77.3$, $R^2 = 0.909$.

a

```
CLUSTAL O(1.2.4) multiple sequence alignment


XP_014588473.2_HSP-1-like      MAPRLGIFLILAGTCIFLQLDHVDGDK-QPITTTLSATMKPDYKCAFPFNYRGKWYFDCT     59
XP_023507327.1_HSP-1-like      MAPRLGIFLILAGTCIFLQLDHVDGDAEQPMGAHPSATVKPENKCVFPFNYRADRYYDCT     60
                               **************************  **: :  ***:**: **.******.. *:***

XP_014588473.2_HSP-1-like      RADSFFKWCSLNEDYSGKWKYCVDDDYAKCVFPFVYRGQTYNRCTTDGSLFWISWCSVTT     119
XP_023507327.1_HSP-1-like      KADSFYHWCSLTEDYRGSWKYCAATDYAKCVFPFVYRGLTYESCTTDGSLFRISWCSVTP     120
                               :****::****.*** *.****.  ************ **: ******** *******

XP_014588473.2_HSP-1-like      SYDRDGAWRYCYNTILNCCTVHLTHGHECNECFEICMKKIKKSQA    164
XP_023507327.1_HSP-1-like      NYDHHGAWKYC----------------------------------    131
                               .**:.***:**
```

b

```
CLUSTAL O(1.2.4) multiple sequence alignment


NP_001075402.1-HSP1      MAPRLGIFLIWAGACIFLQLDHVDGDLQTTGADHSATVNPDQQLIMTKHSATVTPENKCV   60
P81121.1_HSP-1           -------------------------DLQTTGADHSATVNPDQQLIMTKHSATVTPENKCV   35
CAA07074.1_HSP1          --------------------------------------NPDQQLIMTKHSATVTPENKCV   22
                                                               **********************

NP_001075402.1-HSP1      FPFNYRGYRYYDCTRTDSFYRWCSLTGTYSGSWKYCAATDYAKCAFPFVYRGQTYDRCTT   120
P81121.1_HSP-1           FPFNYRGYRYYDCTRTDSFYRWCSLTGTYSGSWKYCAATDYAKCAFPFVYRGQTYDRCTT   95
CAA07074.1_HSP1          FPFNYRGYRYYDCTRTDSFYRWCSLTGTYSGSWKYCAATDYAKCAFPFVYRGQTYDRCTT   82
                         ************************************************************

NP_001075402.1-HSP1      DGSLFRISWCSVTPNYDHHGAWKYC      145
P81121.1_HSP-1           DGSLFRISWCSVTPNYDHHGAWKYC      120
CAA07074.1_HSP1          DGSLFRISWCSVTPNYDHHGAWK--      105
                         ***********************
```

c

```
CLUSTAL O(1.2.4) multiple sequence alignment


CAE46517.1_HSP2          MALQLGVFLIWAGVCIFLQLDHVDGDQQQIVNDHSSTRKPDNKCVFPFKYQGRQYYDCTR  60
NP_001075347.1_HSP-2     MALQLGVFLIWAGVCIFLQLDHVDGDQQQIVNDHSSTRKPDNKCVFPFKYQGRQYYDCTR  60
                         ************************************************************

CAE46517.1_HSP2          TDSFHRWCSLTENYSGKWRYCVAEDYAKCFFPFVYRGRTYHTCTTDGSFFLIPWCSVTPN  120
NP_001075347.1_HSP-2     TDSFHRWCSLTENYSGKWRYCVAEDYAKCFFPFVYRGRTYHTCTTDGSFFLIPWCSVTPN  120
                         ************************************************************

CAE46517.1_HSP2          YDRDGGWKYCMCTVHLTHGHECNEAFEICVKKIKKSQA          158
NP_001075347.1_HSP-2     YDRDGGWKYCM---------------------------          131
                         ***********
```

d

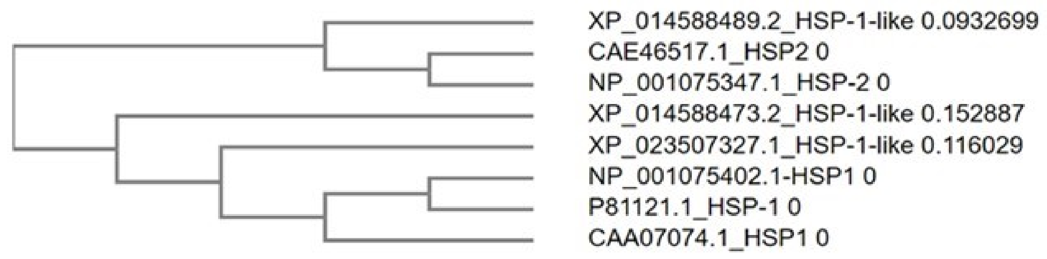

Supplementary Fig. 7 | Proteoforms of HSP-1 (six) and HSP-2 (two) identified from the tryptic peptide sequences (Table 1). a. Two HSP-1 proteoforms were identified from the spots 1, 2, 3, 7, and 25 (e-value=2e-24), showing the same sequence in the common region and different lengths in the common region. **b.** Three HSP-1 proteoforms were identified from the spots 15, 17, 18, 19, and 20 (e-value=2e-31), also with the same sequence in the common region and different lengths. **c.** Two HSP-2 proteoforms were identified from the spot 8 (e-value=1e-5), with the same sequence and different lengths. **d.** Phylogram involving HSPs showed that the HSP-1-like proteoform detected in spot 9 (not shown) is closer to the HSP-2 in spot 8 than the other HSP-1 proteoforms. For spot 9, a unique HSP-1 proteoform, XP_014588489.2 (e-value=1e-5), was recovered. The analysis of identification of the number of gene copies in the reference genome (tBlastn - using NCBIrefseq_genomes database) indicated the access code NC_009153.3 - Chromosome 10 to all proteins identified as HSPs (HSP-1 and HSP-2) (e-value=8e-24 to 9e-31), and also the access code NC_009165.3 - Chromosome 22 to proteins identified as HSP-2 (e-value=3e-07). Blastp (blast.ncbi.nlm.nih.gov/Blast.cgi, database: NCBInr for *Equus caballus*, taxid: 9796, coverage and identity values greater than 70% and 30%, respectively) was used for sequence similarity, and Clustal Omega (ebi.ac.uk/Tools/msa/clustalo/) was used for multiple sequence alignment.

a

```
CLUSTAL O(1.2.4) multiple sequence alignment


XP_014589684.2      MLHLALRTNSMALLPVLLFLAAVLLPFFPASGQDPGFAALSITKSEVQKEIVNKHNDLRR     60
O19010.1            ----------MALLPVLLFLAAVLLPFFPASGQDPGFAALSITKSEVQKEIVNKHNDLRR     50
NP_001075343.1      ----------MALLPVLLFLAAVLLPFFPASGQDPGFAALSITKSEVQKEIVNKHNDLRR     50
                              **************************************************

XP_014589684.2      TVSPLASNMLKMQWDSKTATNAQNWANKCLLQHSKAEDRAVGTMKCGENLFMSSIPNSWS     120
O19010.1            TVSPLASNMLKMQWDSKTATNAQNWANKCLLQHSKAEDRAVGTMKCGENLFMSSIPNSWS     110
NP_001075343.1      TVSPLASNMLKMQWDSKTATNAQNWANKCLLQHSKAEDRAVGTMKCGENLFMSSIPNSWS     110
                    ************************************************************

XP_014589684.2      DAIQNWHDEVHDFKYGVGPKTPNAVVGHYTQVVWYSSYRVGCGIAYCPKQGTLKYYYVCQ     180
O19010.1            DAIQNWHDEVHDFKYGVGPKTPNAVVGHYTQVVWYSSYRVGCGIAYCPKQGTLKYYYVCQ     170
NP_001075343.1      DAIQNWHDEVHDFKYGVGPKTPNAVVGHYTQVVWYSSYRVGCGIAYCPKQGTLKYYYVCQ     170
                    ************************************************************

XP_014589684.2      YCPAGNYVNKINTPYEQGTPCARCPGNCDNGLCTNSCKYEDLVSNCDSLKKIAGCEHELL     240
O19010.1            YCPAGNYVNKINTPYEQGTPCARCPGNCDNGLCTNSCEYEDLVSNCDSLKKIAGCEHELL     230
NP_001075343.1      YCPAGNYVNKINTPYEQGTPCARCPGNCDNGLCTNSCEYEDLVSNCDSLKKIAGCEHELL     230
                    *************************************:**********************

XP_014589684.2      KENCKATCRCENKIY     255
O19010.1            KENCKTTCQCENKIY     245
NP_001075343.1      KENCKATCQCENKIY     245
                    *****:**:******
```

b

```
CLUSTAL O(1.2.4) multiple sequence alignment


NP_001075401.1      MAFLPVVMFLAAVLLPSLPTEGKDPAFSALLTTQTQVQREIVNKHNELRKSVSPPASNML     60
CAA07160.1          ------------------------------------------------------PASNML     6
                                                                          ******

NP_001075401.1      KMEWSREATANAQKWANKCTLEHSSADDRKTSTRCGENIYMSSDPTPWSDAIQSWYDESL     120
CAA07160.1          KMEWSREATANAKRWANKCTLEHSSADDRKTSTRCGENIYMSSDPTPWSDAIQSWYDESL     66
                    ************::**********************************************

NP_001075401.1      DFTYGVGPKSAGSVVGHYTQAVWYSSYRVGCGIAYCPNQESLKYYYVCQYCPVGNNVNKK     180
CAA07160.1          DFTYGVGPKSAGSVVGHYTQAVWYSSYRVGCGIAYCPNQESLKYYYVCQYCPVGNNVNKK     126
                    ************************************************************

NP_001075401.1      NTPYQQGTPCASCPGNCDNGLCTNSCEYEDLLSNCDSLKKTAGCEHELLKEKCKATCRCE     240
CAA07160.1          NTPYEQGHPCASCPGNCDNGICTNSCEYEDLLSNCDSLK---------------------     165
                    ****:** ************:******************

NP_001075401.1      NKIY          244
CAA07160.1          ----          165
```

c

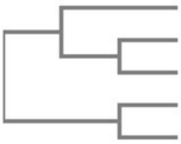

Supplementary Fig. 8 | Proteoforms (five) of CRISP-3 (HSP-3) identified from the tryptic peptide sequences obtained from the spots 50 and 51 (Table 1). a. Three CRISP-3 proteoforms were identified (e-value=8e-17), with different lengths and 99% identity in the common region. **b.** Two CRISP-3 proteoforms were identified (e-value=2e-10), with different lengths and 98,8% identity in the common region. **c.** Phylogram representing these protein sequences corroborated the generation of two groups obtained for the sequence alignment. The number of gene copies recovered in the reference genome (NCBIrefseq_genomes) indicated that the access NC_009163.3 - Chromosome 20 corresponds to the five protein accessions identified. Blastp (blast.ncbi.nlm.nih.gov/Blast.cgi, database: NCBInr for Equus caballus, taxid: 9796, coverage and identity values greater than 70% and 30%, respectively) was used for sequence similarity, and Clustal Omega (ebi.ac.uk/Tools/msa/clustalo/) was used for multiple sequence alignment.

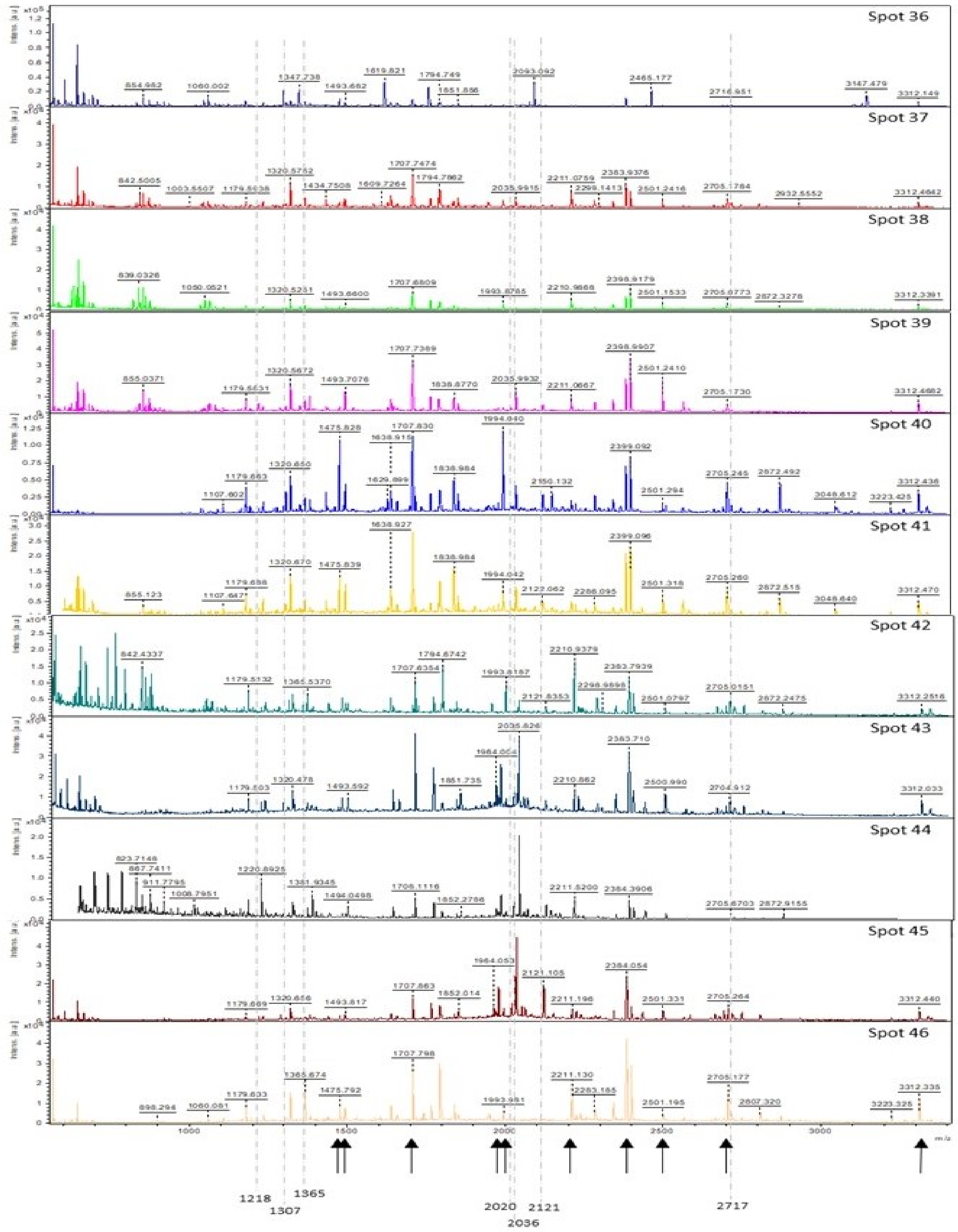

Supplementary Fig. 9 | Mass spectra of the tryptic peptides from kallikrein samples (36-46). Mass spectra of the tryptic peptides from kallikrein samples (36-46). Comparison of all spectra evidenced similar kallikrein peaks (arrows) representing the same peptide, reported in Table 2.

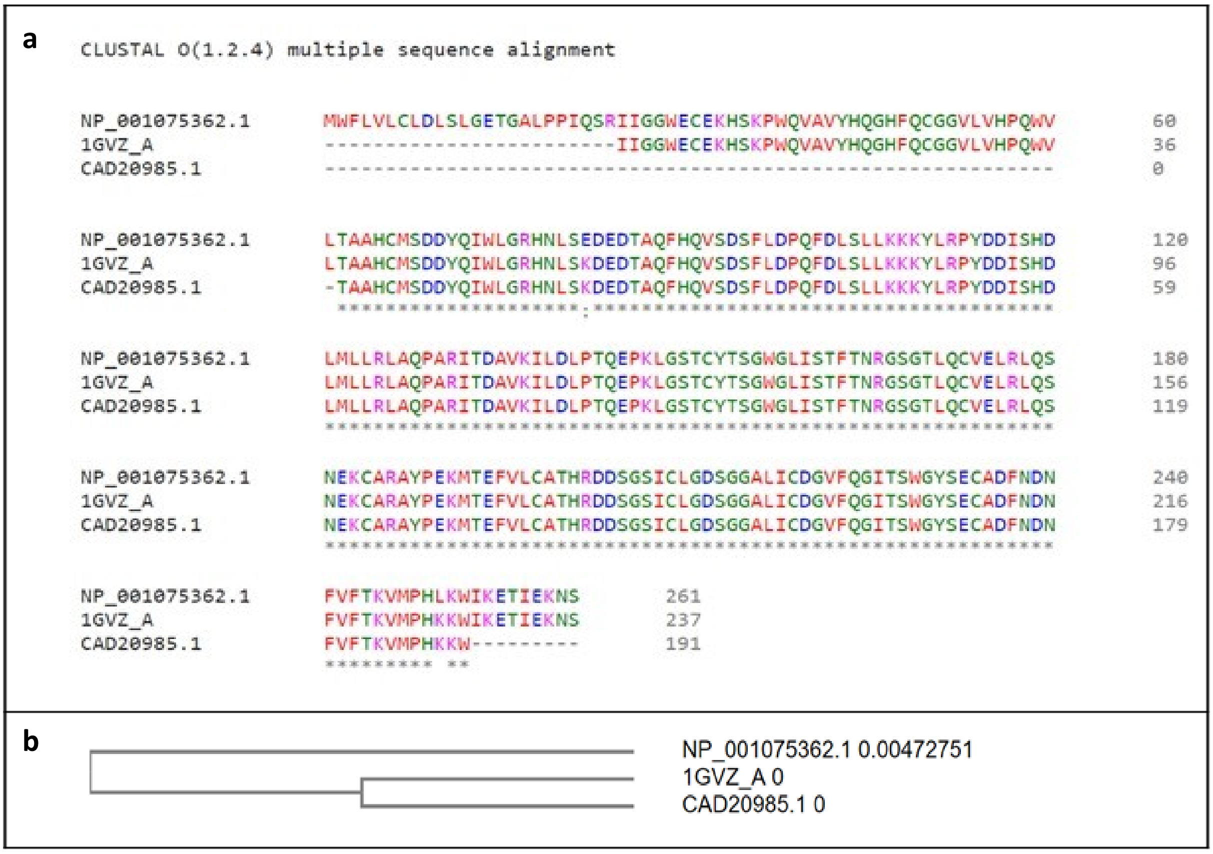

Supplementary Fig. 10 | Proteoforms of kallikreins (KLK) identified from the tryptic peptide sequences obtained from the spots 39, 41, 44, and 45 (Table 1). a. The three KLKs proteoforms were identified (e-value=5e-14 to 9e-16), with different lengths and 98.8% identity in the common region. **b.** Phylogram representing these protein sequences indicated greater proximity between 1GVZ_A and CAD20985.1. Analysis of the number of copies of the genes, based on the tryptic peptide sequences obtained from the spots (NCBIrefseq_genomes) recovered nine accesses, with coverage and identity greater than 70% and 30%, respectively (e-value=e-5 to 100e-55): NC_009145.3, NC_009146.3, NC_009147.3, NC_009150.3, NC_009153.3, NC_009155.3, NC_009156.3, NC_009162.3, and NC_009170.3, described on chromosomes 2, 3, 4, 7, 10, 12, 13, 19 and 27, respectively. Blastp (blast.ncbi.nlm.nih.gov/Blast.cgi, database: NCBInr for Equus caballus, taxid: 9796, coverage and identity values greater than 70% and 30%, respectively) was used for sequence similarity, and Clustal Omega (ebi.ac.uk/Tools/msa/clustalo/) was used for multiple sequence alignment.

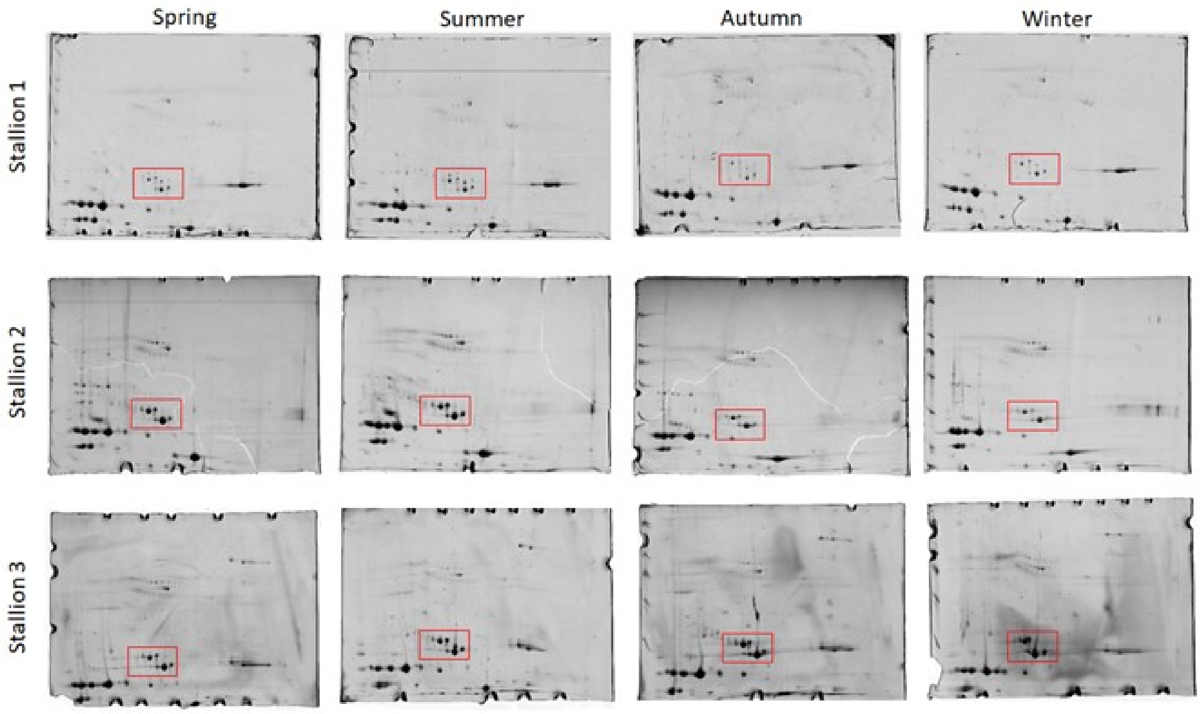

Supplementary Fig. 11 | Additional information for Figure 4a. Full-length gels obtained by two-dimensional electrophoresis analysis of seminal plasma from stallions during different seasons. The red boxes highlight the spots identified as kallikreins in **Figure 4a**.
